# Engineered viral DNA polymerase with enhanced DNA amplification capacity: a proof-of-concept of isothermal amplification of damaged DNA

**DOI:** 10.1038/s41598-020-71773-6

**Published:** 2020-09-14

**Authors:** Carlos D. Ordóñez, Ana Lechuga, Margarita Salas, Modesto Redrejo-Rodríguez

**Affiliations:** 1 Centro de Biología Molecular Severo Ochoa, Consejo Superior de Investigaciones Científicas and Universidad Autónoma de Madrid, CSIC-UAM, Madrid, Spain; 2 Department of Biochemistry, Universidad Autónoma de Madrid and Instituto de Investigaciones Biomédicas “Alberto Sols”, CSIC-UAM, Madrid, Spain

**Keywords:** DNA, Protein design

## Abstract

The development of whole genome amplification (WGA) and related methods, coupled with the dramatic growth of sequencing capacities, has changed the paradigm of genomic and genetic analyses. This has led to a continual requirement of improved DNA amplification protocols and the elaboration of new tailored methods. As key elements in WGA, identification and engineering of novel, faithful and processive DNA polymerases is a driving force in the field. We have engineered the B-family DNA polymerase of virus Bam35 with a C-terminal fusion of DNA-binding motifs. The new protein, named B35-HhH, shows faithful DNA replication in the presence of magnesium or an optimised combination of magnesium and manganese divalent cofactors, which enhances the replication of damaged DNA substrates. Overall, the newly generated variant displays improved amplification performance, sensitivity, translesion synthesis and resistance to salt, which are of great interest for several applications of isothermal DNA amplification. Further, rolling-circle amplification of abasic site-containing minicircles provides a proof-of-concept for using B35-HhH for processive amplification of damaged DNA samples.

## Introduction

DNA polymerases (DNAPs) are ubiquitous enzymes that act as guardians of the genetic information in all biological replicons across the three domains of life, in addition to viruses and other mobile genetic elements^1^. Moreover, they are a key tool for DNA-based applications in biotechnology and biomedicine. Naturally occurring DNAPs can be phylogenetically and functionally classified into 8 families, which represent their evolutionary divergence for specialised molecular processes in DNA metabolism, including genome replication and an array of repair mechanisms^2,3^. Thus, highly faithful and processive replicative enzymes (replicases) and usually associated with a proofreading activity, belong to families A, B, C and D, whereas retrotranscriptases (including telomerase) are specialised for copying RNA into DNA. Members from families X and Y, as well as the recently added primases-polymerases (PrimPols)^4^, are mostly distributive enzymes that display different ranges of solvent-exposed active sites, allowing them to participate in a variety of challenging DNA repair pathways where they tolerate aberrant DNA features, albeit at the expense of a very low polymerisation accuracy^5^.

During the last 60 years, the extensive biochemical characterisation of numerous A- and B-family DNA replicases has facilitated their use as tools for many applications in molecular biology and biotechnology^6,7^. Prominent among these is bacteriophage Φ29 DNA polymerase (Φ29DNAP)^8^, a B-family DNAP characterised by a high fidelity and an outstanding processive DNA synthesis ability, coupled with an intrinsic strand displacement capacity. These features enabled the development of the so-called MDA or Multiple Displacement Amplification method^9^, which uses random hexamer primers and allows the amplification of DNA substrates without prior sequence information. Other important advantages of MDA over PCR can be summarised as follows: (1) any DNA substrate can be amplified exponentially from very low amounts—in the femto or attogram range; (2) the amplicons formed using Φ29DNAP are much larger than those obtained by PCR; and (3) the technology can be established in almost any laboratory as no specific platform is required to perform amplification reactions isothermally at 30 ºC. A further development of this methodology is its recent application in whole genome amplification (WGA) of very low input amounts, down to the single-cell level^10^.

Notwithstanding the above advantages, the application of MDA for single-cell WGA has some limitations, including inherent amplification bias, particularly of GC-rich sequences^11,12^. Amplification artifacts can also arise, leading to chimeric formation of false structural variants as well as allelic dropout where one allele is differentially amplified over the other in a heterozygous sample, which is a major obstacle for the clinical application of single-cell WGA^13^.

The presence of modified bases in DNA samples blocks DNA synthesis by most DNAPs and thus strongly impairs DNA amplification of damaged DNA from environmental or ancient DNA^14,15^. Those shortcomings, along with the recent exponential growth of sequencing capacities, have motivated the development of new DNA amplification methods that have been nourished by novel and tailored DNAPs with enhanced proficiency and a variety of improved features, including thermostability, resistance to inhibitors or even amplification of “reluctant” samples^6,16,17^. Among the new-engineered DNAPs, protein fusions with single- or double-stranded DNA-binding proteins have been successfully created for improved MDA and PCR applications^18–21^. Fusion of the same DNA-binding domains can improve^22^ but also hinder^23^ DNA polymerase activity of different DNAPs.

In line with the expanding repertoire of DNAPs suitable for DNA amplification, we have recently characterised a DNAP from tectivirus Bam35 (B35DNAP)^24^ as a new B-family faithful replicase that shares some features with Φ29DNAP, including high processivity and strand displacement capacity. Moreover, B35DNAP is endowed with intrinsic translesion synthesis (TLS) capacity, allowing it to replicate abasic site-containing templates without insertion/deletion mutations. We successfully improved B35DNAP proficiency by adding a C-terminal fusion of two Helix-hairpin-Helix [(HhH)_2_] DNA binding motifs^20,21^. We show here that this new engineered variant, hereafter termed B35-HhH, promotes faithful DNA replication similar to the parental enzyme, in the presence of either magnesium (Mg^2+^) or an optimised combination of both Mg^2+^ and manganese (Mn^2+^) divalent cofactors. We also analysed the substrate specificity of TLS of B35DNAP and B35-HhH, finding that both can bypass abasic sites and also oxidative base modifications. However, only B35-HhH was able to partially overcome bulkier modifications such as thymine dimers, likely because this damage induces major structural changes in the DNA helix that strongly obstruct DNA replication. Overall, the newly generated variant displays improved amplification performance, sensitivity, TLS and resistance to salt, which will be of great interest for several applications of isothermal DNA amplification.

## Materials and methods

### DNA polymerase proteins

**Figure 1:**
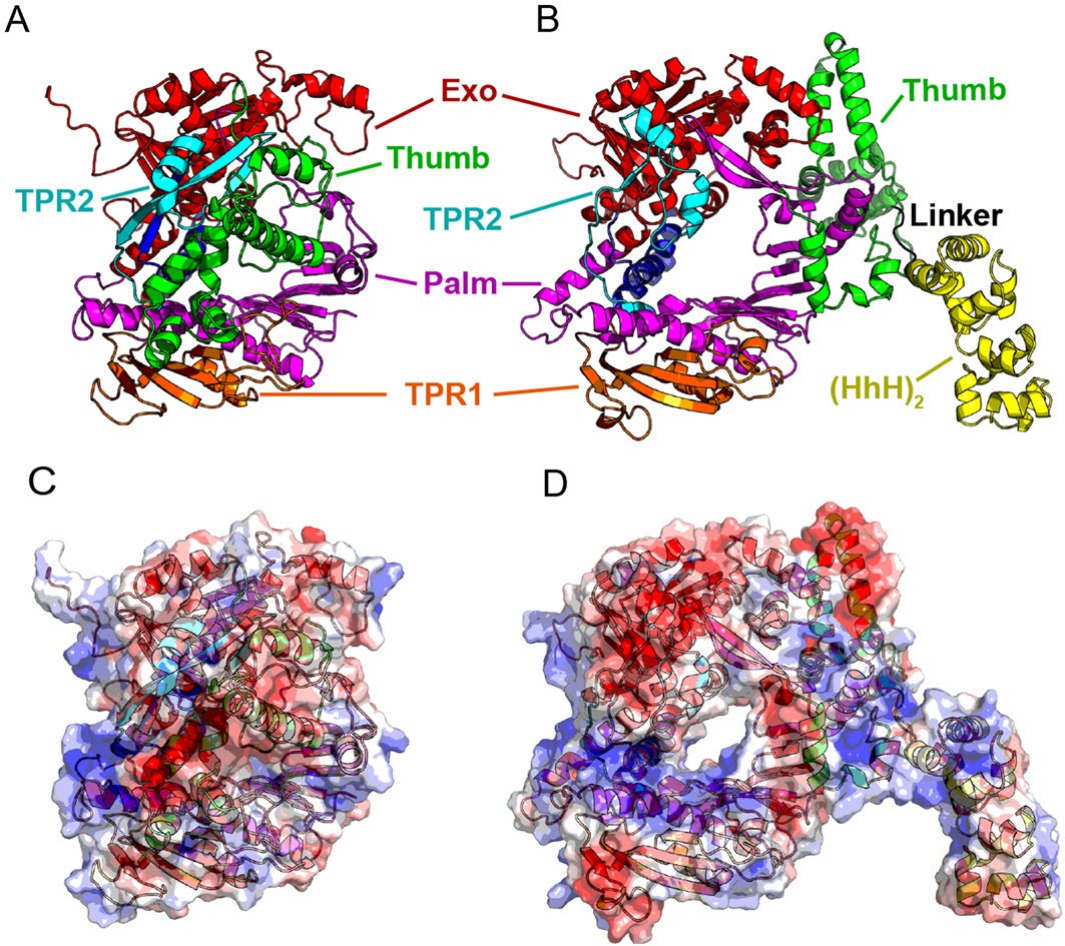
Structural models of B35DNAP and B35-HhH proteins. Representation of models of B35DNAP (**A**) and B35-HhH (**B**) variants, generated with the Robetta server^49^ and further optimised with GalaxyRefine^50^. Models were rendered with PyMOL Molecular Graphics System (Schrödinger, LLC). Surface electrostatic potentials of wild-type and B35-HhH proteins were obtained with the Adaptive Poisson-Boltzmann Solver (APBS) plugin for PyMOL (**C**,**D**).

The B35-HhH expression plasmid was constructed from the expression vector of the Φ29-HI engineered variant of Φ29DNAP (plasmid pJLw2-HI)^20^. Briefly, the B35DNAP sequence was amplified from the pT7.4::B35 DNAP plasmid^24^ using the primers B35HindIII_fw and B35KasI_rev (Table S1) and cloned into the pJLw2-HI backbone previously digested with HindIII and KasI and gel-excised. This new vector codes for a fusion protein comprising B35DNAP, a short linker (GTGSGA) and C-terminal His-tagged H and I DNA-binding domains of *Methanopyrus kandleri* topoisomerase V (Fig. 1)^20^. The complete B35DNAP coding sequence was verified by Sanger sequencing.

B35-HhH was expressed in *Escherichia coli* BL21(DE3) using ZYM-5052 autoinduction medium^25^, supplemented with 100 mg/mL ampicillin. Cultures were harvested after 16.5 h at 25ºC and cells were disrupted by grinding with alumina and suspended in buffer A (50 mM Tris–HCl, pH = 7.5, 7 mM β-mercaptoethanol, and 5% [v/v] glycerol) containing 0.5 M NaCl. Alumina and cell debris were removed by centrifugation at 2,000 rpm for 5 min and an additional centrifugation at 12,000 rpm for 20 min was performed to obtain a cell-free protein extract. High-speed centrifugation steps were performed in a Sorvall RC-5B Refrigerated Superspeed Centrifuge using a fixed angle GSA rotor at 4ºC. Absorbance (260 nm) of the supernatant was adjusted to 120 U/mL before DNA precipitation in the presence of 0.3% polyethyleneimine and 1 M NaCl. After centrifugation at 12,000 rpm for 10 min, proteins in the supernatant were precipitated by the addition of ammonium sulphate to 69% saturation and centrifugation at 12,000 rpm for 30 min. The pellet was then washed with 25 mL of buffer A supplemented with ammonium sulphate (65%), resuspended in 10 volumes of buffer A and applied to a Ni–NTA agarose resin column (Qiagen N.V.). The column was washed with increasing concentrations of imidazole in buffer A containing 0.5 M NaCl, and fractions containing B35-HhH, spanning 40–200 mM imidazole, were applied to a phosphocellulose column (P11; Whatman). After washing with increasing concentrations of NaCl, the eluted fractions with the purified protein (0.4–0.5 M NaCl) were combined and applied to a heparin agarose column (Sigma). Finally, after extensive washing with buffer A supplemented with 0.2 M NaCl, the purified DNAP was eluted with buffer A at 1 M NaCl, dialysed against buffer A, containing 50% glycerol, 0.5 M NaCl and 0.025% Tween 20 and maintained at – 20 or – 70 ºC for long-term storage.

Parental B35DNAP protein was obtained from the laboratory stock and purified as detailed previously^24^.

### Nucleotides and DNAs

Highly pure dNTPs were supplied by GE Healthcare [γ-^32^P]ATP and [α-^32^P]dATP (3,000 Ci/mmol) were purchased from PerkinElmer. PAGE-purified oligonucleotides (Table S1) were synthesised by Sigma or IDT. As indicated in Table S1, some oligonucleotides contained modified bases, abbreviated as follows: tetrahydrofuran (THF) as an abasic site analog, cyclobutane thymine dimer (T:T) or thymine glycol (Tg).

### Primer extension assays

DNA oligonucleotides (Table S1) were 5′-labeled with [γ-^32^P]ATP (PerkinElmer) and T4 polynucleotide kinase (New England Biolabs). Template/primer duplexes were generated with oligonucleotide OL15 hybridised to complementary 33 mer oligonucleotides (OL33c, OL33c-F, OL33c-T:T, OL33c-t) to generate the primer-extension substrates, as depicted in the corresponding figures. When indicated, OL15, OL17, OL19 and OL33 were also labelled and loaded as size markers.

Assays were performed in a final volume of 20 µL containing 50 mM Tris–HCl (pH 7.5), 1 mM DTT, 4% (v/v) glycerol, 0.1 mg/mL BSA, 0.05% (v/v) Tween 20 and, unless otherwise stated, 1 nM of the indicated 5′-labelled primer/template duplex, 10 nM DNAP, and the indicated concentration of dNTPs. Negative controls without DNAP contained the highest dNTP concentration used in each experiment.

Reactions were initiated by the addition of either 10 mM MgCl_2_, 1 mM MnCl_2_ or 1 mM CoCl_2_, as indicated and, after incubation for 10 min or the indicated times at 37ºC, were stopped by adding 10 µL formamide loading buffer (98% formamide, 20 mM EDTA, 0.5% [w/v] bromophenol blue, and 0.5% [w/v] xylene cyanol). Samples were analysed by denaturing 7 M urea-20% PAGE in Tris–Borate-EDTA buffer (TBE). Gel bands were detected using a phosphorimager (Typhoon FLA 7,000) and processed with ImageJ 1.52 k (https://image.nih.gov/ij) software (NIH)^26^.

**Figure 2:**
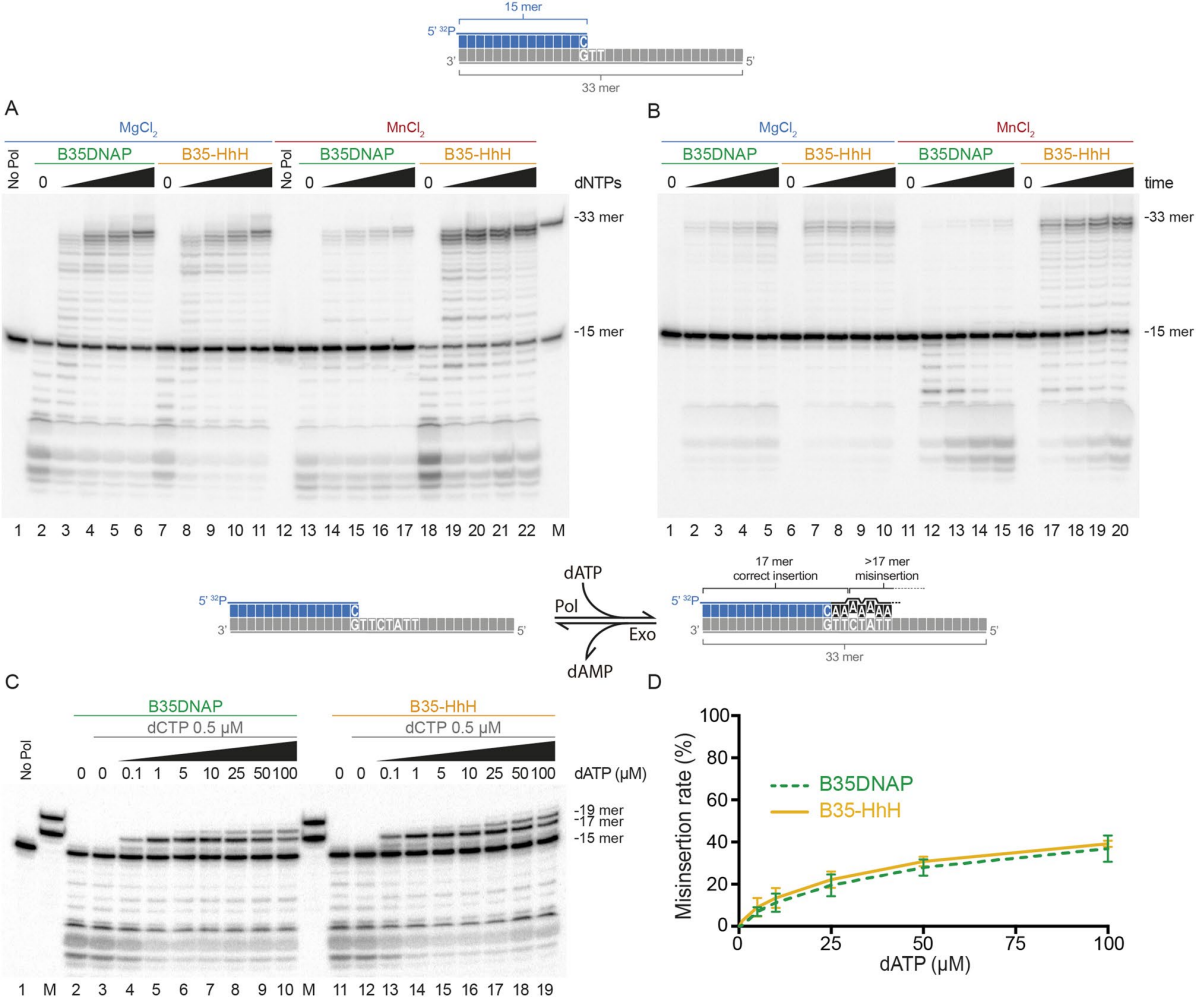
Faithful DNA proofreading and polymerase activities of engineered B35-HhH DNA polymerase. Primer extension assays with an oligonucleotide template/primer duplex substrate (1 nM) as depicted above. In the scheme, the template is represented in grey, primer in blue and incorporated nucleotides during the primer extension in black. Reactions were performed in the presence 10 nM of the indicated DNA polymerase and increasing concentration of dNTPs—25, 50, 100 and 500 nM (**A**), or in a time-course experiment during 15, 30, 60 or 120 s with 100 nM dNTPs (**B**). Nucleotide insertion fidelity was analysed on running extension assays triggered with 10 mM MgCl_2_ (**C**). 15-, 17- and 19-mer or 15 and 33-mer oligonucleotides (OL15, OL17, OL19 and OL33, Table S1) were loaded as size markers (lane M). Relative misinsertion rate at each dATP concentration (**D**) was determined from three independent experiments. See “Materials and methods” for details.

Misincorporation frequency in running-start extension assays was analysed as described^27^, with minor modifications. The same primer/template substrate described above was incubated with the indicated DNAP and increasing concentrations of only dATP, complementary to positions + 1, + 2, + 4 and + 6 were added. Thus, as depicted in Fig. 2C, 16- and 17-mer bands correspond to the correct insertion of dAMP, whereas further extension implies one or more A:A mismatch formation and extension. The reaction mixture also contained 500 nM dCTP to reduce the 3′-5′ degradation of the primer terminus without interfering with the insertion capacity. The misincorporation ratio was calculated as relative incorrect/total nucleotide insertion from three independent experiments.

### Rolling circle amplification assays

Genomic M13mp18 single-stranded circular DNA (laboratory stock)^28^ was annealed to oligonucleotide OL-M13 (Table S1) in a buffer containing 0.2 M NaCl and 60 mM Tris–HCl, pH 7.5. The M13 rolling circle amplification (RCA) reaction was performed at 37ºC in a final volume of 75 µL with 1.5 nM primed M13mp18 ssDNA, 40 µM dNTPs, 50 nM of the indicated DNAP, 0.04 µCi/µL [α-^32^P]dATP and 10 mM MgCl_2_. Samples (23 µL) were withdrawn at the indicated times and the reaction was stopped by the addition of a final concentration of 30 mM EDTA and 0.5% SDS. The λ DNA ladder used as a size marker was obtained by labelling genomic λ DNA with Klenow fragment (New England Biolabs) in the presence of [α-^32^P]dATP ^29^. The reaction products were analysed by electrophoresis in alkaline 0.7% agarose, detected by phosphor imaging (Typhoon FLA 7,000) and processed with ImageJ 1.52 k (https://image.nih.gov/ij) software (NIH)^26^.

Circular ssDNA substrates, without or with one or two discrete abasic sites, were generated by circularisation of MC, MC-1F and MC-2F oligonucleotides (Table S1). Primer extension assays on linear substrates were also performed for reference (Fig. S1). RCA substrates (minicircles) were generated with CircLigase (Epicentre). Non-circularised molecules were degraded with Exonuclease I (New England Biolabs) and samples were purified with the QIAquick PCR Purification Kit (Qiagen N.V.) (Fig. S2). Circularised ssDNA substrates were quantified by band densitometry after denaturing electrophoresis (7 M Urea, 8% PAGE, 1X TBE) and Ethidium Bromide staining, using a pattern of increasing amounts of oligonucleotide as reference. Finally, singly-primed substrates were generated by hybridisation with the MCc-7 primer (Table S1). RCA was carried out in a final volume of 10 µL with 24 nM of the indicated primed DNA minicircle, 200 µM dNTPs, 50 nM of the indicated DNAP, 220 nM *E. coli* SSB (Enzymatics Inc.), 2 µCi [α-^32^P]dATP and the indicated metal cofactor(s). The reactions were stopped after 18 h of incubation at 37 ºC and processed as indicated above.

### Multiple displacement amplification assays and fidelity analysis

Two different DNA plasmids, pUC19 (Thermo Fisher Scientific, 50% GC content) and pIJ702 (Addgene #35287, 70% GC), were used for isothermal MDA. MDA assays and fidelity analyses were carried out as described^24^. In brief, unless otherwise stated the incubation mixture (20 μL) contained 10 mM MgCl_2,_ 40 mM Tris·HCl, pH 7.5, 50 mM KCl, 0.025% Tween 20, 500 μM dNTPs, 25 μM 3′-protected hexamer random primers (IDT), 50 nM B35DNAP or B35-HhH, the indicated amount of plasmid and 10 nM MnCl_2_ (when shown). The reactions were incubated for 16 h at 37 °C, after which 4 μL of each amplification product was digested with EcoRI-HF for 2 h at 37 ºC and subjected to electrophoresis in 0.7% agarose gels. The DNA was detected by GreenSafe Premium (NZYTech).

Quantification of MDA amplification products was performed with AccuBlue High Sensitivity dsDNA Quantitation Kits (Biotium), using the FLUOstar Optima microplate reader. Three independent MDA experiments with 1 ng of initial plasmid DNA input were carried out and both input and reaction products were quantified in parallel for calculation of DNA amplification rate. The difference between B35DNAP and B35-HhH amplification performance was tested by a paired t-test (Prism GraphPad Software).

To analyse the fidelity of B35DNAP, we treated a fraction of the amplified DNA product with EcoRI-HF and DpnI (New England Biolabs) for 2 h at 37 °C. The digested products were purified using the QIAquick PCR Purification Kit (Qiagen N.V.), and 170 ng of pUC19 linear plasmid was circularised with T4 DNA ligase (New England Biolabs) in a final volume of 100 μL. To analyse mutations in the lacZα gene, we transformed 20 ng of ligated DNA into the *E. coli* Top10F’ strain (Invitrogen) for blue/white colony screening. Several LacZα mutants (white colonies) were further analysed by plasmid DNA sequencing with primer LacZ_sec (Table S1). The background mutation frequency (f0) for each experiment was determined with the original plasmid digested with EcoRI-HF, purified, and re-ligated under the same conditions.

## Results

### B35-HhH, a proficient Bam35 DNA polymerase variant.

Wild-type DNA polymerase from tectivirus Bam35 (B35DNAP)^24^ is a highly processive replicase. As is the case for Φ29DNAP, this processivity is likely defined by a high stability of the DNAP/DNA complex by virtue of a ring-shaped, positively charged, internal structure formed by thumb, palm, TPR1, and TPR2 subdomains^30,31^. Indeed, Φ29DNAP and B35DNAP variants lacking the TPR2 motif show a drastically diminished processivity^24,31^.

Multiple sequence alignments and structure predictions indicated that the thumb of B35DNAP is much larger (137 amino acids) than that of Φ29DNAP (45 amino acids), and it is also predicted to be richer in alpha helices. This would likely play a role in the interaction with the upstream DNA duplex. Structural models suggested that the thumb might be folded towards the DNA-interacting core in the apoprotein (Fig. 1A,C), likely through contact with the TPR2 subdomain as well as electrostatic interactions with the electropositive DNA binding groove.

The aforementioned differences in the polymerisation domain between B35DNAP and Φ29DNAP make the outcomes of protein engineering for its DNA polymerisation capacity unpredictable. That notwithstanding, we elected to generate a variant with a C-terminal (HhH)_2_ fusion (Fig. 1B), equivalent to the engineered Φ29DNAP, which showed an improvement in DNA amplification over the wild-type enzyme (Φ29-HI)^20^. The structural model of the new fusion protein, B35-HhH, showed a substantial conformation rearrangement of the TPR2 and thumb subdomains (Fig. 1B,D).

We purified this new variant, B35-HhH, and performed a detailed characterisation alongside the parental DNAP. First, to analyse the polymerisation and exonuclease activities of the protein, we performed primer extension assays on a labelled 15-mer oligonucleotide annealed to the 33-mer template using an increasing concentration of dNTPs (Fig. 2A) or progressive incubation times (Fig. 2B). Wild-type B35DNAP activity was initially characterised in the presence of Mg^2+^ as a metal cofactor^24^, which allows faithful and processive DNA replication by family-B DNAPs^32^. To characterise in detail the new variant, we tested both DNAPs using either Mg^2+^ or Mn^2+^ as a cofactor. The presence of Mn^2+^ commonly stimulates DNA polymerisation and proofreading activity of some DNAPs, although it typically reduces selectivity for the formation of correct base pairs^33–36^.

In the presence of Mg^2+^ (Fig. 2A), both DNAPs generated the same DNA products, which indicated a similar coordination between 5′-3′ DNA polymerisation and 3′–5′ proofreading activities. Thus, in the absence of dNTPs, we could detect exonucleolytic degradation products (lanes 2 and 7) but increasing the concentration of dNTPs resulted in effective elongation of the primer, with very similar patterns (lanes 3–6, 8–11). Time-course experiments indicated a slightly enhanced DNA synthesis by B35-HhH in shorter reaction times in the presence of Mg^2+^ (Fig. 2B, lanes 2–5 vs. 7–10). Strikingly, B35DNAP showed reduced and delayed exonuclease and polymerase activities in Mn^2+^-triggered reactions, whereas B35-HhH activity was enhanced by this cofactor (lanes 13–17 vs. 18–22 in Fig. 2A and lanes 12–15 vs. 17–20 in Fig. 2B).

As mentioned above, a key feature in replicative DNAPs is the fidelity during DNA replication. To assess the DNA replication fidelity of B35-HhH, we first analysed the stable incorporation of incorrect nucleotides during polymerisation by a misincorporation assay (see “Materials and methods”), analysing the misinsertion in running start conditions in the presence of increasing concentrations of dATP and a fixed concentration of the 3′-terminal nucleotide (dCTP), to lessen exonucleolytic degradation of the primer (see “Materials and methods” for details). In the presence of Mg^2+^, the ratio of misincorporated dAMP (incorrect/correct) was very similar by both DNAPs, even at very high dATP concentrations (Fig. 2C,D). However, a clear stimulation on DNA synthesis and misincorporation was detected in the case of B35-HhH when other divalent ions, such as Co^2+^ or Mn^2+^ was used (Fig. S3A).

Overall, these results show that B35-HhH has coordinated exonuclease and DNA polymerisation capacity and primer extension fidelity, with a very similar balance between the two activities to the parental enzyme when Mg^2+^ was used as cofactor. However, B35HhH showed a greater stimulation by Co^2+^ or, particularly, Mn^2+^ ions (Fig S3A), which may be due to specific role of metal cofactors in (HhH)_2_ DNA binding capacity^37^.

### Translesion synthesis capacity of B35DNAP and B35-HhH

B35DNAP is endowed with abasic site TLS capacity opposite to abasic sites^24^. We thus tested B35-HhH TLS capacity opposite to a tetrahydrofuran (THF) template and compared this capacity with that of the parental protein. To further characterise the spectra of DNA base modifications that might be amplified using these DNAPs, we analysed TLS of both enzymes opposite to different replication-blocking DNA damage products, including a thymine-glycol (Tg) oxidised base and cyclobutane thymine dimers (T:T), and also the effect of different divalent ions as a cofactor.

**Figure 3:**
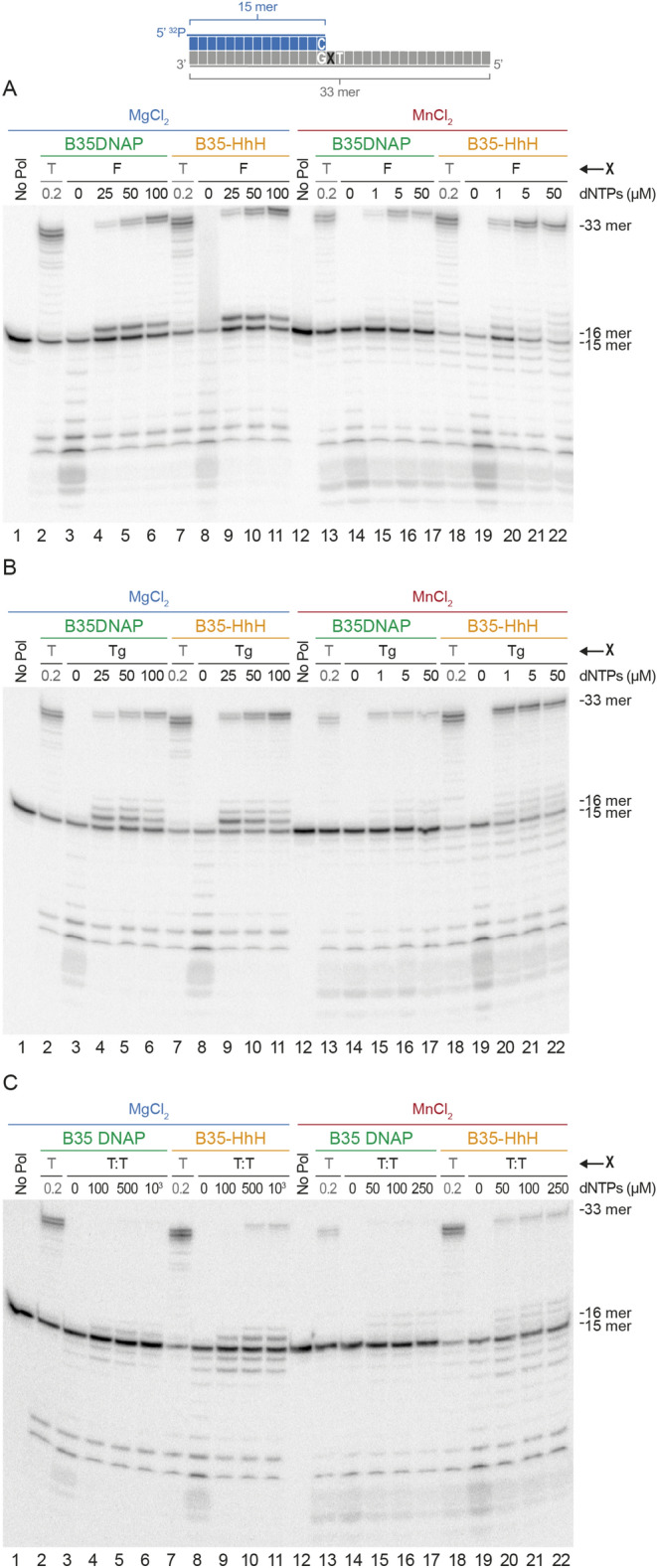
Substrate specificity of TLS capacity of B35DNAP and B35-HhH. Primer extension analysis opposite to damage-containing templates. Reactions were incubated for 10 min at 37ºC in the presence of the indicated dNTPs concentrations and were triggered either with 10 mM MgCl_2_ or 1 mM MnCl_2_, as indicated. Base indicated with an X in the diagram corresponds to a tetrahydrofurane abasic site analog (F) in A, thymine-glycol (Tg) in B and ciclobutane thymine dimers (T:T) in C.

To do this, we performed primer extension assays using an increasing concentration of dNTPs and using a template with a damaged base in the first position + 1 (Fig. 3). In the presence of Mg^2+^ ions, undamaged templates as well as abasic site and Tg-containing substrate duplexes were replicated with a similar balance of degradative and synthetic capacities, as they showed the same dependence on dNTPs concentration (lanes 1–11 in Fig. 3A, B). By contrast, T:T resulted in a strong blockage that impeded primer extension by B35DNAP, yet this could be bypassed to some extent by the B35-HhH variant irrespective of the metal cofactor (Fig. 3C). However, TLS was overall enhanced in the presence of Mn^2+^ as cofactor, with a notable reduction of the pause opposite to the damaged base by both DNAPs, which is the limiting step for this capacity^24^. Nevertheless, replication of damaged templates was again more efficient with B35-HhH than with the parental enzyme in the presence of Mn^2+^ (lanes 12–22 in Fig. 3A–C). Also, in agreement with the aforementioned effect of Mn^2+^ ions on DNA polymerization (Fig S3A), the B35-HhH stimulation of TLS ability by Mn^2+^ was greater than the other cofactors tested as Mg^2+^ or Co^2+^ (Fig. S3B).

Altogether, these experiments indicate that B35-HhH is a proficient replicase and, similar to B35DNAP, it is endowed with DNA polymerisation and proofreading activity, as well as TLS capacity. However, contrary to the parental enzyme, B35-HhH polymerisation activity was stimulated in the presence of Mn^2+^ or Co^2+^. The higher enhancement of the TLS ability and the strong stimulation of the B35-HhH by Mn^2+^ makes it an attractive candidate to damaged DNA amplification by Helix-harpin-helix-fused DNAPs.

### Rolling circle and multiple displacement DNA amplification by B35-HhH

**Figure 4:**
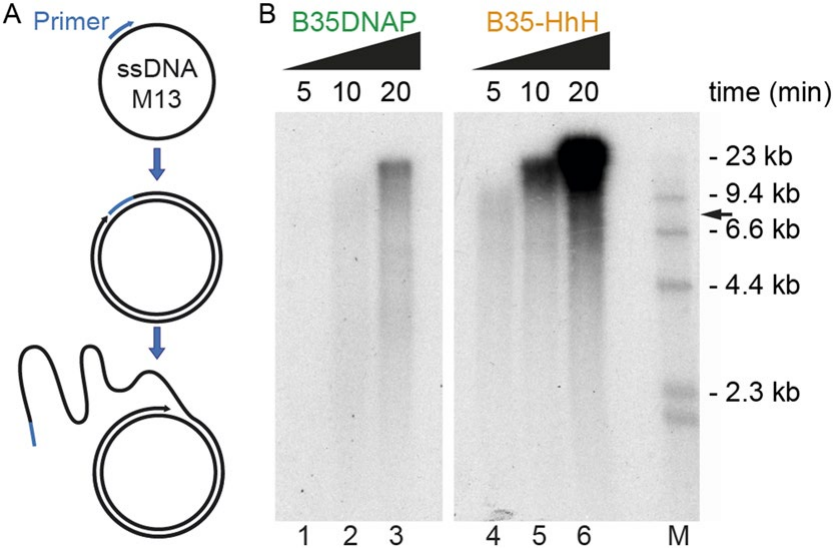
Rolling circle amplification of circular DNA by B35-HhH. (**A**) Schematic representation of the rolling circle amplification (RCA) process of a singly primed M13 DNA, which can give rise to a large single-stranded DNA product due to the intrinsic strand displacement capacity. (**B**) Alkaline gel electrophoresis of DNA products synthesised by B35DNAP and B35-HhH in RCA assays. λ-HindIII DNA ladder was used as a size marker (lane M). Full-length M13 DNA size is indicated with a black arrow.

We also tested the effect of the (HhH)_2_ domains in processive replication of circular DNA substrates. First, we performed RCA of singly-primed M13 DNA, which allowed us to analyse processivity and strand-displacement capacity of the DNAPs (Fig. 4A). As expected, assays with both enzymes gave rise to a replication product larger than full-length M13 DNA (7.25 kb), indicating strand displacement capacity (Fig. 4B). The absence of intermediate products also suggested high replication processivity. Notably, compared with the parental enzyme, B35-HhH synthesised a larger amount of final product and at a faster replication rate (lanes 4–6 vs 1–3).

**Figure 5:**
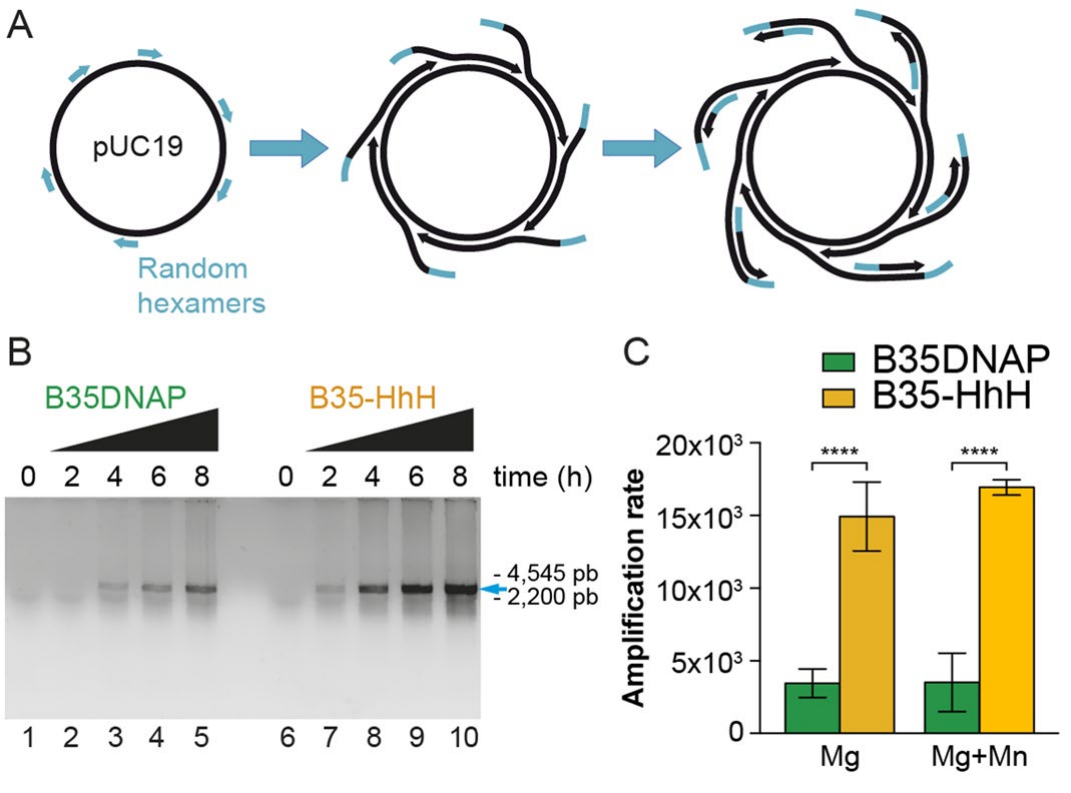
Highly proficient multiple displacement amplification of plasmid DNA by B35-HhH. Isothermal multiple displacement amplification of plasmid circular DNA is initiated by random hexamers that prime overlapping processive DNA replication events, giving rise to multibranched DNA structures (**A**) that can be digested and analysed by non-denaturing TAE agarose electrophoresis (**B**). Amplification rate by B35DNAP and B35-HhH was determined by quantification of input and reaction product (**C**) as detailed in “Materials and methods”.

As mentioned earlier, isothermal MDA allows the amplification of very low amounts of plasmidic DNA in the presence of random hexamers as primers, without prior knowledge of the sample DNA sequence (Fig. 5A). This reaction can be carried out only by highly processive DNAPs, including B35DNAP^38^. We found that B35-HhH was threefold more efficient than B35DNAP for this reaction and it could amplify the pUC19 plasmid in a shorter time than the parental enzyme (Fig. 5B,C).

**Figure 6:**
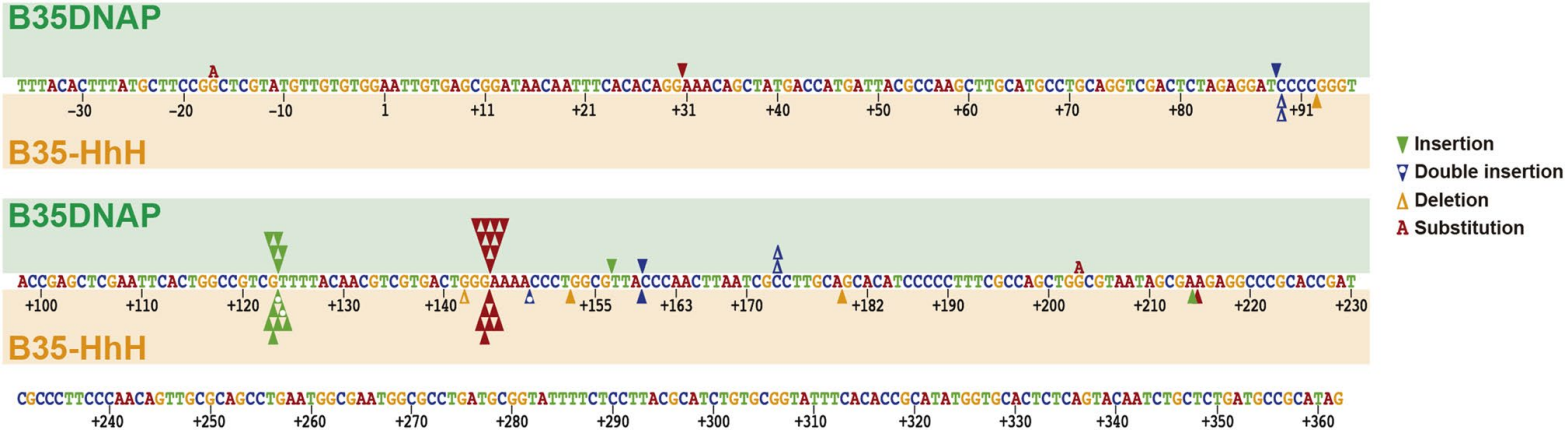
Spectra of single base changes by B35DNAP and B35-HhH in DNA amplification experiments. Bases are colored in red (A), blue (C), green (T), and orange (G). Base insertions, deletions, and substitutions by B35DNAP or B35-HhH are indicated above and below the sequence, respectively, according to the legend, as inverted triangles (▼), greek letter delta (Δ), and the mutated base, respectively, maintaining the same color code. Position 1 is the first transcribed nucleotide of the lacZ gene.

We also analysed the fidelity of random-primed MDA reactions by white/blue colony screening (see “Materials and methods”), using Top10 cells, which have been reported to have a lower background mutation ratio in these experiments^39^. Under the conditions assayed, B35DNAP amplified plasmids with an error rate of 1.06 × 10^–5^, similar to our previous results using XL-1 cells^24^, and about 3.5-fold higher than B35-HhH (2.76 × 10^–6^) (Table 1A). Further, 45 mutant (white) colonies (23 from samples amplified with B35DNAP and 22 from B35-HhH) were sequenced to determine the most frequent type of error. We found that both DNAPs introduced a very low proportion of base substitutions and deletions, with insertions on homopolymeric tracts being the most common cause of mutated (white) colonies (Fig. 6). This is also in accord with our previous analysis of B35DNAP DNA amplification fidelity^24^.

**Table 1:** Determination of error rate values for B35DNAP and B35-HhH during MDA of plasmid DNA in the presence of Mg^2+^ (A) or Mg^2+^/Mn^2+^ (B) ions.

Sample	Experiment	Colonies		f	d	Error rate (× 10^–6^)
*A. Cofactor: MgCl*_*2*_* 10 mM*						
Control	1	9,894		0.18%		
	2	15,480		0.08%		
	3	8,496		0.56%		
	4	6,980		0.62%		
	5	1608		0.56%		
B35DNAP	1	6,598		2.88%	11.88	6.64
	2	10,248		2.84%	11.54	7.00
	3	5,380		4.63%	11.97	9.93
	4	3,010		6.18%	12.14	13.40
	5	4,176		6.66%	10.97	16.26
B35-HhH	1	17,441		0.72%	14.05	1.13
	2	13,511		0.68%	13.84	1.27
	3	6,065		0.99%	13.91	0.89
	4	3,631		2.26%	13.42	3.58
	5	9,462		3.89%	14.02	6.94
Average	B35DNAP			4.64%	11.70	10.64 ± 3.71
	B35-HhH			1.71%	13.85	2.76 ± 2.30
*B. Cofactor: MgCl*_*2*_* 10 mM* + *MnCl*_*2*_* 10 nM*						
Control	4	6,980		0.62%		
	5	1608		0.56%		
B35DNAP	4	2,938		6.06%	12.27	12.97
	5	3,868		5.95%	11.03	14.27
B35-HhH	4	3,450		1.19%	14.08	1.19
	5	4,551		3.69%	14.02	6.53
Average	B35DNAP			4.00%	7.77	13.62 ± 0.65
	B35-HhH			1.63%	9.36	3.86 ± 2.67

Mutant frequencies (f) were determined by dividing the total number of white plaques by the total number of plaques. Template doublings (d) were calculated using the equation d = log_2_((ng product)/(ng input)). Background frequency (f0) was calculated in control experiments with plasmid DNA purified from bacteria, digested and re-ligated in the same conditions (“Materials and methods”). Error rate was calculated using the equation ER = (f − f0)/bp·d, where bp is the number of detectable sites in the lacZ gene (342 bp).

As mentioned, Mn^2+^ ions normally reduce the Km for nucleotides in DNAPs, which might result in enhanced TLS but also in a greater number of misincorporations, as well as reduced processivity^32,36^. Thus, it would be expected that the presence of Mn^2+^ hampers faithful and processive DNA synthesis. We attempted to optimise the presence of metal cofactors in the MDA reaction to improve amplification of damaged samples without hindering the amplification yield and the fidelity of the reaction. Approximately 10 nM MnCl_2_ could be used in MDA without interfering with the amplification yield (Fig. S4). Importantly, similar to the standard conditions (10 mM MgCl_2_, Fig. S5), when the reaction mixture was supplemented with 10 nM MnCl_2_, the B35-HhH variant polymerised a ~ fourfold higher amount of amplified DNA product than parental B35DNAP (Fig. 5C) and with a very similar error rate (Table 1B).

Therefore, the simultaneous addition of 10 mM MgCl_2_ and 10 nM MnCl_2_ provided a balanced condition with a higher yield and faithful DNA amplification that could be tested to amplify damaged samples by MDA.

### Successful amplification of challenging DNA samples

**Figure 7:**
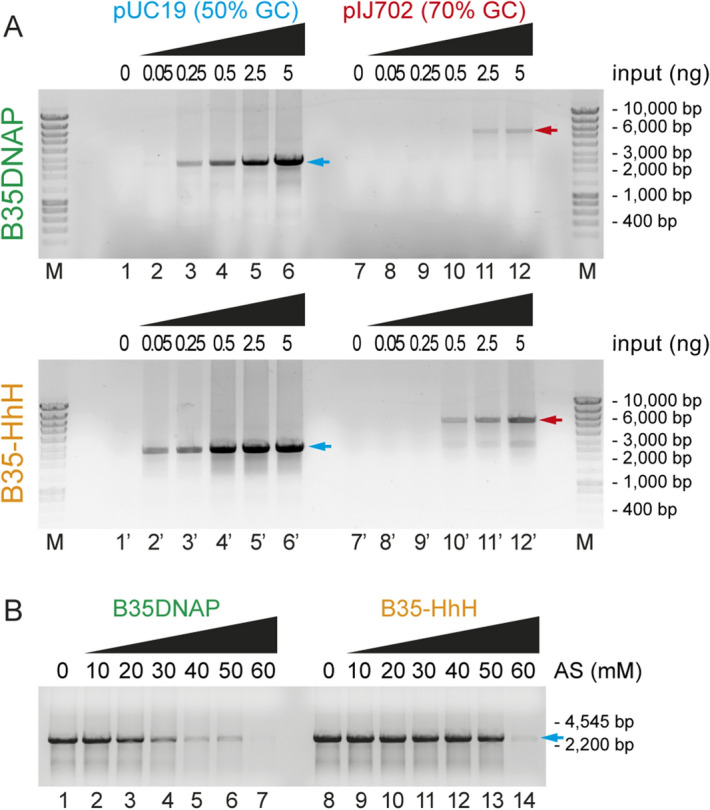
Isothermal multiple displacement amplification in different conditions. Multiple displacement amplification of plasmid DNA with different GC content (**A**) and in the presence of high salt concentrations (**B**). Plasmid full-length size after EcoRI digestion is indicated with blue or red arrow for pUC19 and pIJ702, respectively.

One of the main aims for improving processive DNAPs is to overcome the amplification barrier of difficult samples, including those with high GC content or the presence of inhibitors such as high ionic strength^21,40^. We thus tested the enzymes for the amplification of plasmid DNA with high GC content (pIJ702, 70% GC content) and in the presence of increasing ammonium sulphate concentrations. As shown in Fig. 7A, B35-HhH could amplify plasmid DNA with high GC content with less input DNA and to a higher extent than the parental DNAP, suggesting that the engineered variant may amplify complex DNA with less bias for GC content in the DNA.

Also, DNA amplification by B35-HhH was successful in the presence of ammonium sulphate up to 50 mM (Fig. 7B, lane 13), whereas B35DNAP activity was impaired at this ionic strength, showing a strongly reduced amplification capacity from 30 mM (Fig. 7B, lane 4). This suggests that B35-HhH is suitable for amplification of DNA samples without prior purification steps.

Overall, these results demonstrate that fusion of DNA-binding domains to B35DNAP improves the sensitivity for DNA amplification, with increased performance even on GC-rich sequences and tolerance to inhibitory conditions such as high ionic strength.

### Rolling circle amplification of damaged DNA

**Figure 8:**
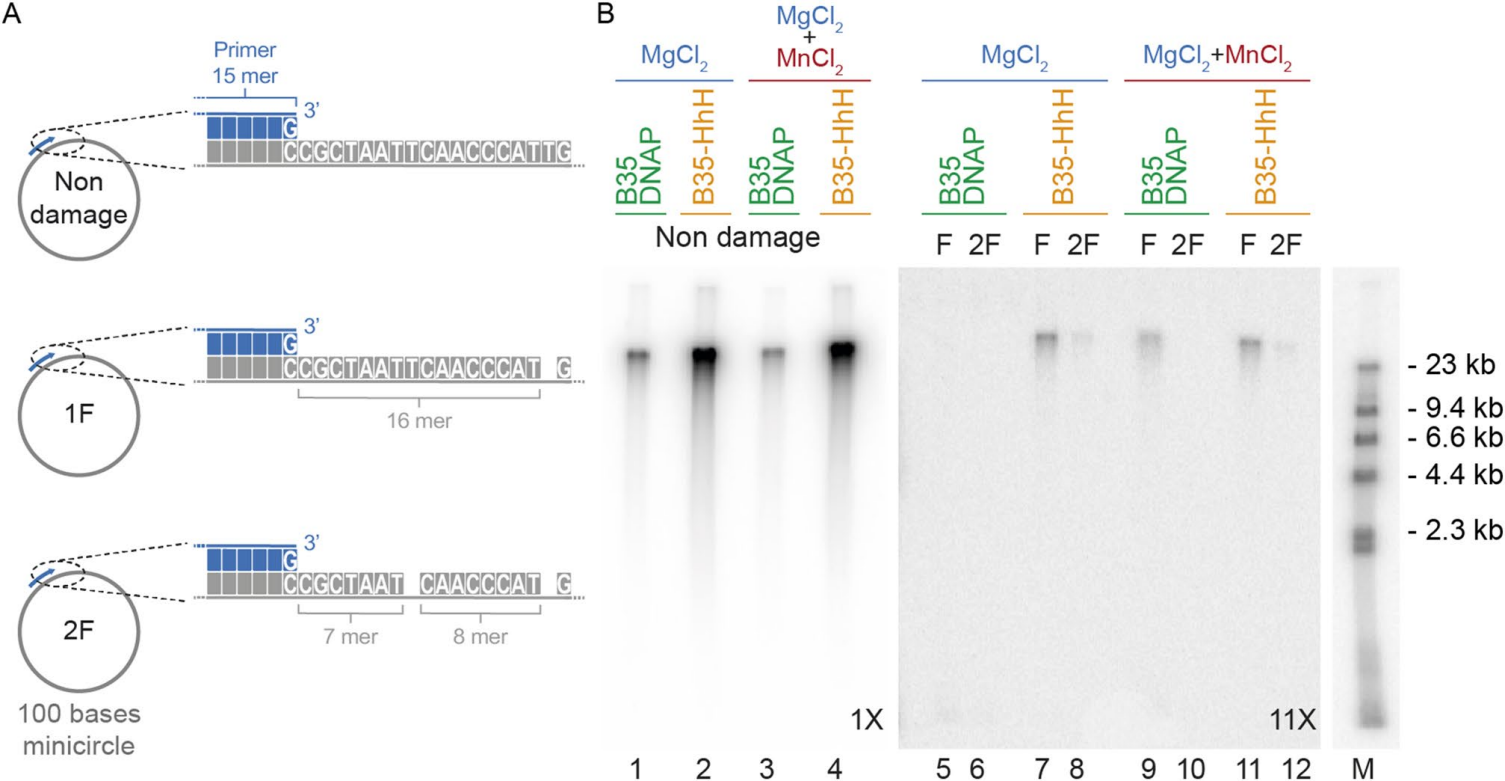
Rolling circle amplification of DNA containing abasic sites. (**A**) Schematic representation of primed single-stranded DNA minicircles containing abasic sites. (**B**) Alkaline electrophoresis of minicircle RCA by B35DNAP and B35-HhH. As indicated, reactions were triggered by 10 mM MgCl_2_ plus/minus 10 nM MnCl_2_. DNA form λ bacteriophage was used as a DNA ladder (see “Materials and methods” for details). Panels correspond to a single gel with different ranges of detection in the linear grey scale; i.e. the image was taken with a maximum level and a windowing of 12,283 (1 ×, lanes 1–4), 2,362 (5 ×, for lane M) and 1,092 (11 ×, lanes 5–12) in ImageJ (https://image.nih.gov/ij) software (NIH)^26^.

The successful development of methods to amplify DNA containing base modifications requires the ability to copy, full-length, every molecule with damage, which can be very difficult to assess in a complex sample. Thus, to test the capacity of B35-HhH to amplify damaged DNA, we generated synthetic substrates containing discrete abasic sites, comprising 100 base-length minicircles of single-stranded DNA with one (1F) or two (2F) tetrahydrofuran abasic site analogs (Fig. 8A). The THF analogs were located 5 bases away from each other, thus beyond the mismatch short-term memory of B35DNAP (≤ 4 nt) that counteracts TLS capacity^24^. Moreover, we checked that distance between primer and first abasic site was not affect to the bypass by a primer extension assay by using two different primers (Fig. S1). Given that Mn^2+^ can stimulate the TLS capacity of B35DNAP and B35-HhH, we performed the amplification with 10 mM MgCl_2_, in the presence/absence of 10 nM MnCl_2_, a concentration that did not affect the amplification yield (Fig. 5C) or the fidelity (Table 1).

As shown in Fig. 8B, amplification of damaged DNA by B35DNAP was undetectable when only Mg^2+^ was provided as a metal cofactor (lanes 5–6) and only the substrate with a single abasic site could be amplified in the presence of both Mg^2+^ and Mn^2+^ ions (lane 9). By contrast, B35-HhH could amplify the circle with one abasic site (lanes 7 and 11) and, albeit to a lesser extent, also the substrate with two abasic sites (lanes 8 and 12) both with Mg^2+^ and with Mg^2+^ and Mn^2+^.

Thus, B35-HhH is able to processively replicate abasic site-containing DNA substrates. Altogether, these results constitute a proof-of-concept for the use of B35-HhH or related DNAPs for WGA of damaged DNA.

## Discussion

### B35-HhH, an engineered DNA polymerase with improved amplification proficiency

During the last 60 years, many fields related to genetics and molecular biology have greatly benefited from the characterisation of innumerable DNAPs. Further, for many applications, those DNAPs featuring optimal properties have been engineered from natural enzymes by means of several directed evolution approaches^6,17^. Among them, fusions of single- or double-stranded DNA-binding domains have often improved DNA amplification proficiency of DNAPs in PCR and MDA protocols^18,20,41,42^. Most likely, as has been already suggested, future development of new methods of WGA, rapid detection of DNA or new sequencing technologies will require specialised, tailored DNAPs suitable for specific purposes^7^.

In the present study, we successfully generated an improved version of the B-family DNAP from Bam35 virus (B35DNAP)^24^. The new variant, B35-HhH, has enhanced DNA amplification capacity, and overall similar DNA replication fidelity and TLS capacity to the parental DNAP. Moreover B35-HhH shows improved amplification of plasmidic DNA with very high GC content and also resistance to high ionic strength (Fig. 7). The fusion of two DNA-binding domains has been previously shown to increase efficiency against GC-rich templates in PCR by thermostable DNAPs^18^, but the effect on isothermal DNA amplification of those samples was unclear. Moreover, tolerance to salt can correlate with the ability to function in the presence of other common DNA contaminants, such as heparin and urea^40^. Therefore, B35-HhH-based isothermal amplification of DNA is a novel protocol that provides high DNA yield and assures successful amplification of “reluctant” DNA samples, such as those with high GC content, damaged bases or extraction-derived contaminants.

We also found that, contrary to the parental enzyme, the DNA replication capacity of B35-HhH is enhanced in the presence of Mn^2+^ or Co^2+^ as a metal cofactor, both on damaged and undamaged templates. Further, although Mn^2+^ may reduce faithful and processive DNA replication, we optimised the conditions (10 mM MgCl_2_ plus 10 nM MnCl_2_) for DNA replication stimulated by Mn^2+^ to preserve the fidelity and high-yield replication. On the other hand, despite the effect of Co^2+^ ions were not deeply analysed in this work, it gave rise to a specific stimulation of B35-HhH, with only moderate increasing of the misincorporation. That activation by Co^2+^ may be of interest in non-damaged DNA replication or amplification studies. In line with this, it should be noted that the DNA-binding capacity of the HhH motifs is mediated by metal ions^37^, among which Mn^2+^ has been shown to be bound with greater affinity than Mg^2+^^43^, and our results suggest that Co^2+^ may have a similar effect. Thus, we speculate that the enhanced DNA polymerisation capacity of B35-HhH in the presence of Mn^2+^ and Co^2+^ ions can be due to a role in HhH motif-mediated DNA-binding capacity rather than deriving from a kinetic effect on the DNA polymerisation reaction per se, particularly in our optimised conditions, that is, in the presence of a higher concentration of Mg^2+^ that could be bound at the catalytic centre of the DNAP.

### Towards a WGA method for damaged DNA samples

Genetic analysis or sequencing of DNA samples from environmental sources for research or forensic purposes is often limited by the quality of DNA samples. Environmentally-damaged samples can contain degraded DNA fragments (double-stranded breaks in the DNA), as well as single-stranded nicks and DNA lesions, including abasic sites, alkylated or oxidised bases or pyrimidine dimers^15,44^. Likewise, purified DNA from biological specimens preserved as formalin-fixed tissues very often contains highly fragmented DNA and protein-DNA crosslinks that strongly hinder amplification or analysis. Despite many efforts in the development of improved extraction, repair and DNA amplification protocols, achieving complete DNA amplification remains a major challenge for extensively damaged samples^45–47^. While some of the recent advances in this context entail targeted amplification of known markers^48^, application of WGA methods is currently out of reach.

Forthcoming methods for complete amplification should combine the processivity and fidelity of available DNAPs with a reliable TLS capacity. We show here that B35DNAP and B35-HhH are faithful and processive DNAPs and, moreover, they are able to overcome replication of DNA with replication-blocking lesions, such as oxidised bases or abasic sites. Engineered B35-HhH is superior to the parental enzyme in processivity, amplification rate and the amount of product DNA. The formulation of an optimised combination of Mg^2+^ and Mn^2+^ reported here might be of interest for the development of novel DNA amplification methods for damaged substrates by HhH-based engineered DNAPs. In this scenario, RCA of abasic site-containing minicircles provides a proof-of-concept for the development of novel WGA methods for damaged DNA samples.

## Supplementary information

Supplementary file
