# Supplementary material for: Engineered viral DNA polymerase with enhanced DNA amplification capacity: a proof-of-concept of isothermal amplification of damaged DNA
Source: Sci Rep. 2020 Sep 14;10:15046. doi: 10.1038/s41598-020-71773-6 (PMC7490695; doi:10.1038/s41598-020-71773-6)
Supplement: Supplementary file 1 — Supplementary file [file 41598_2020_71773_MOESM_ESM.pdf]

Engineered viral DNA polymerase with enhanced DNA amplification capacity. A proof-of-concept of isothermal amplification of damaged DNA.

Carlos D. Ordóñez[1], Ana Lechuga[1], Margarita Salas[1#] and Modesto Redrejo-Rodríguez[1,2*]

[1]Centro de Biología Molecular Severo Ochoa, Consejo Superior de Investigaciones Científicas y Universidad Autónoma de Madrid, CSIC-UAM, Madrid, Spain.

[2]Department of Biochemistry, Universidad Autónoma de Madrid and Instituto de Investigaciones Biomédicas "Alberto Sols", CSIC-UAM, Madrid, Spain.

*Correspondence to modesto.redrejo@uam.es

SUPPLEMENTARY INFORMATION

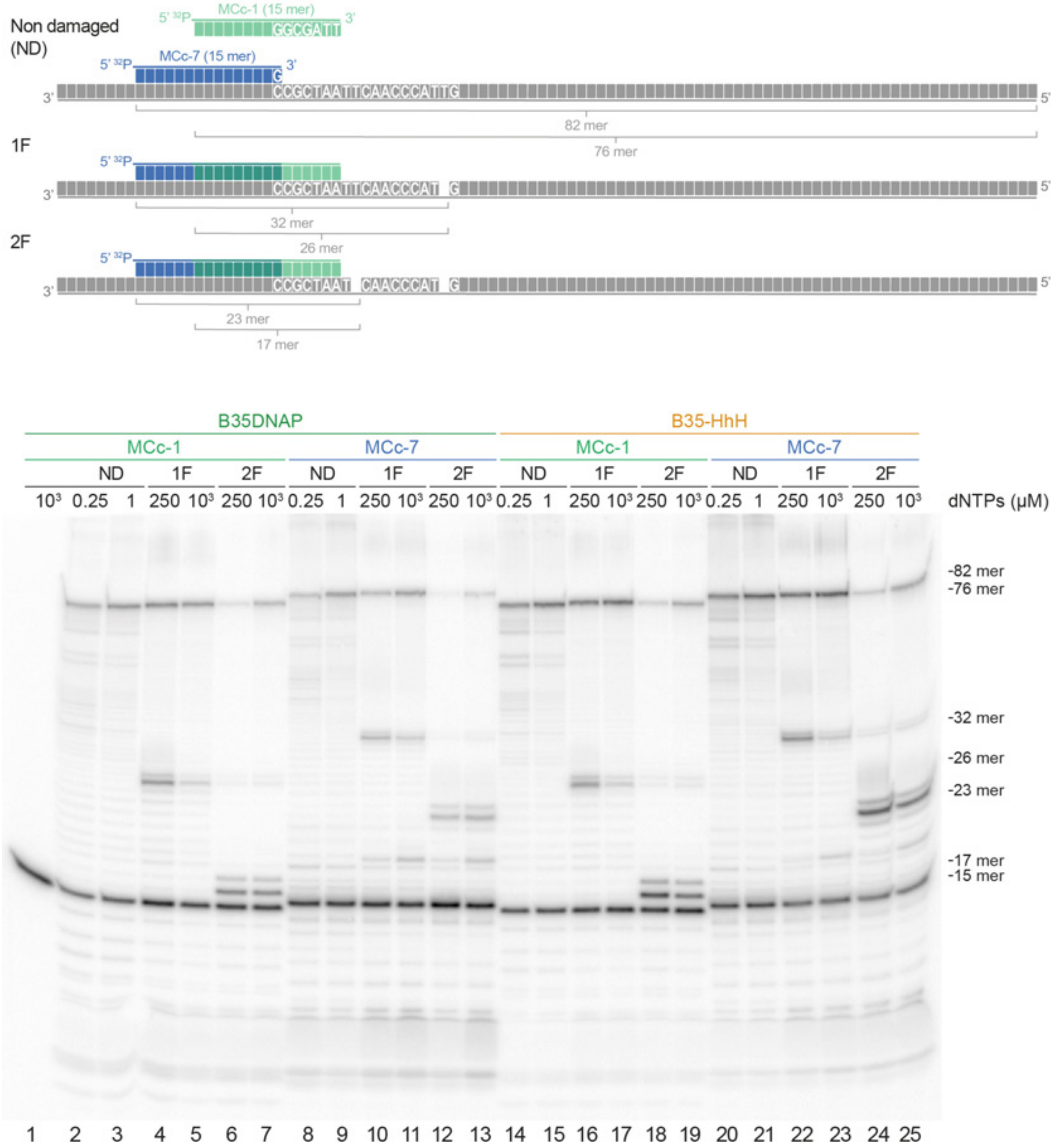

Figure S1. Bypass of abasic sites in minicircle-oligonucleotide substrates.
Control primer extension experiment of translesion synthesis with linear oligonucleotides used for minicircle generation prior to circularisation. Two primers are used, MCc-7 hibridized to 7 nucleotides the first Abasic site in 2F template and MCc-1 in a closer distance. Abasic site analog is indicated in the diagram by the absence of the box that represents nitrogenate bases. The reaction was carried out in presence of 10 mM $MgCl_2$.

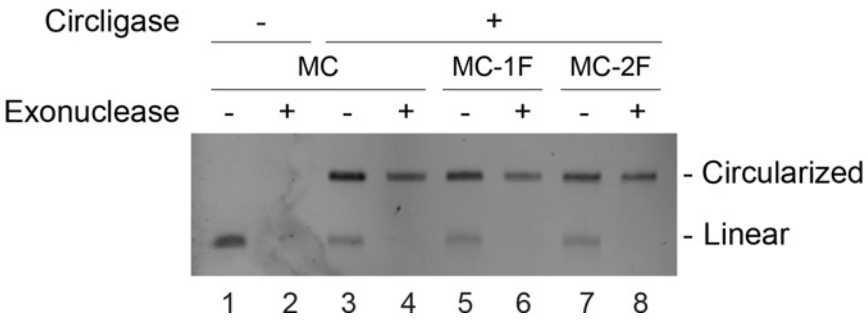

Figure S2. Minicircle ligation.
Denaturing electrophoresis gel where oligonucleotides MC, MC-1F and MC-2F were ligated and treated or not with exonuclease. Exonuclease I degrades linear DNA. As a control oligonucleotide, MC is also shown in the absence of ligation where all the molecules are linear (lane 1) or degraded (lane 2).

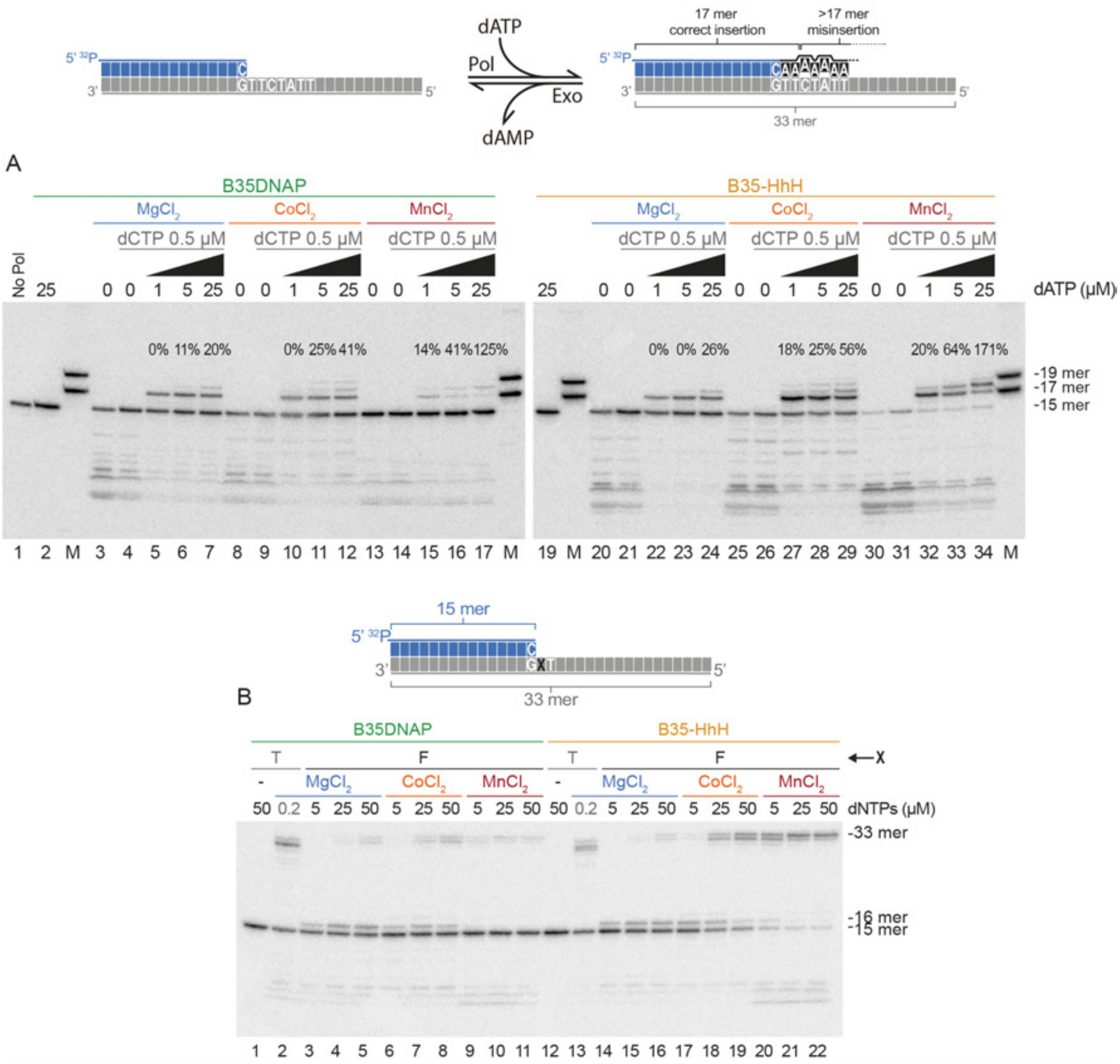

Figure S3. Effect of different metal cofactor in correct nucleotide insertion and Translesion Synthesis.

Denaturing PAGE analysis of primer extension assays with an oligonucleotide template/primer duplex substrate as described in Materials and Methods. In the schemes above each panel, the template is represented in grey, primer in blue and incorporated nucleotides during the primer extension in black. Reactions were incubated for 10 min at 37ºC in the presence of the indicated dNTPs concentrations, the optimum concentration of each salt (10 mM $MgCl_2$, 1 mM $CoCl_2$ or 1 mM $MnCl_2$) and were triggered with 1 nM substrate. Nucleotide insertion fidelity was analysed on running extension with the indicated contraction of dCTP and increasing concentrations of dATP in A. Relative misinsertion rate at each dATP concentration is indicated on each lane. 17- and 19-mer oligonucleotides (OL15, OL17, OL19 and OL33, Table S1) were loaded as size markers (lane M). Base indicated with an X in the diagram corresponds to a tetrahydrofurane abasic site analog (F) in B.

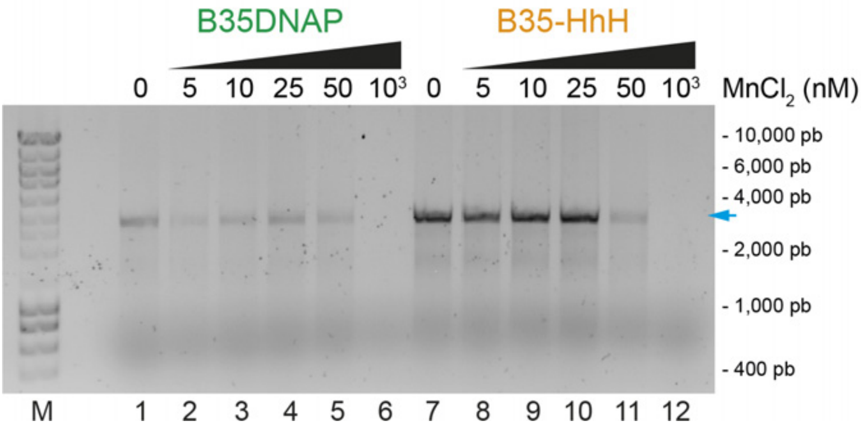

Figure S4. Effect of addition of different concentrations $MnCl_2$ to multiple displacement amplification.

Multiple displacement amplification of pUC19 with 10 mM $MgCl_2$ as is described in Materials and Methods, but complemented with the indicated concentration of $MnCl_2$. Plasmid full-length size after EcoRI digestion is indicated with blue arrow.

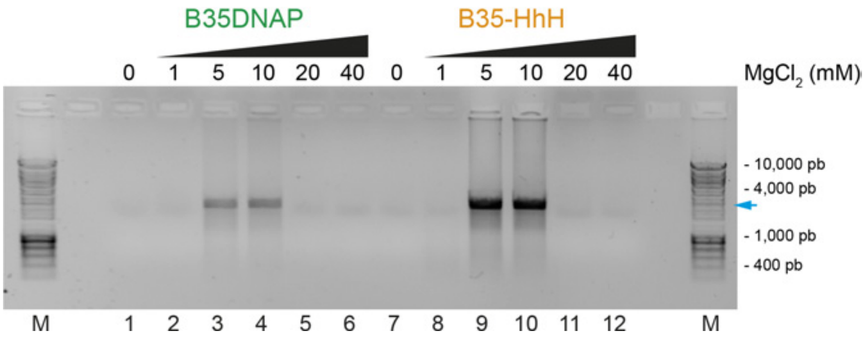

Figure S5. Effect of $MgCl_2$ concentration to multiple displacement amplification. Multiple displacement amplification of pUC19 with the indicated $MgCl_2$ concentration. Plasmid full-length size after EcoRI digestion is indicated with blue arrow.

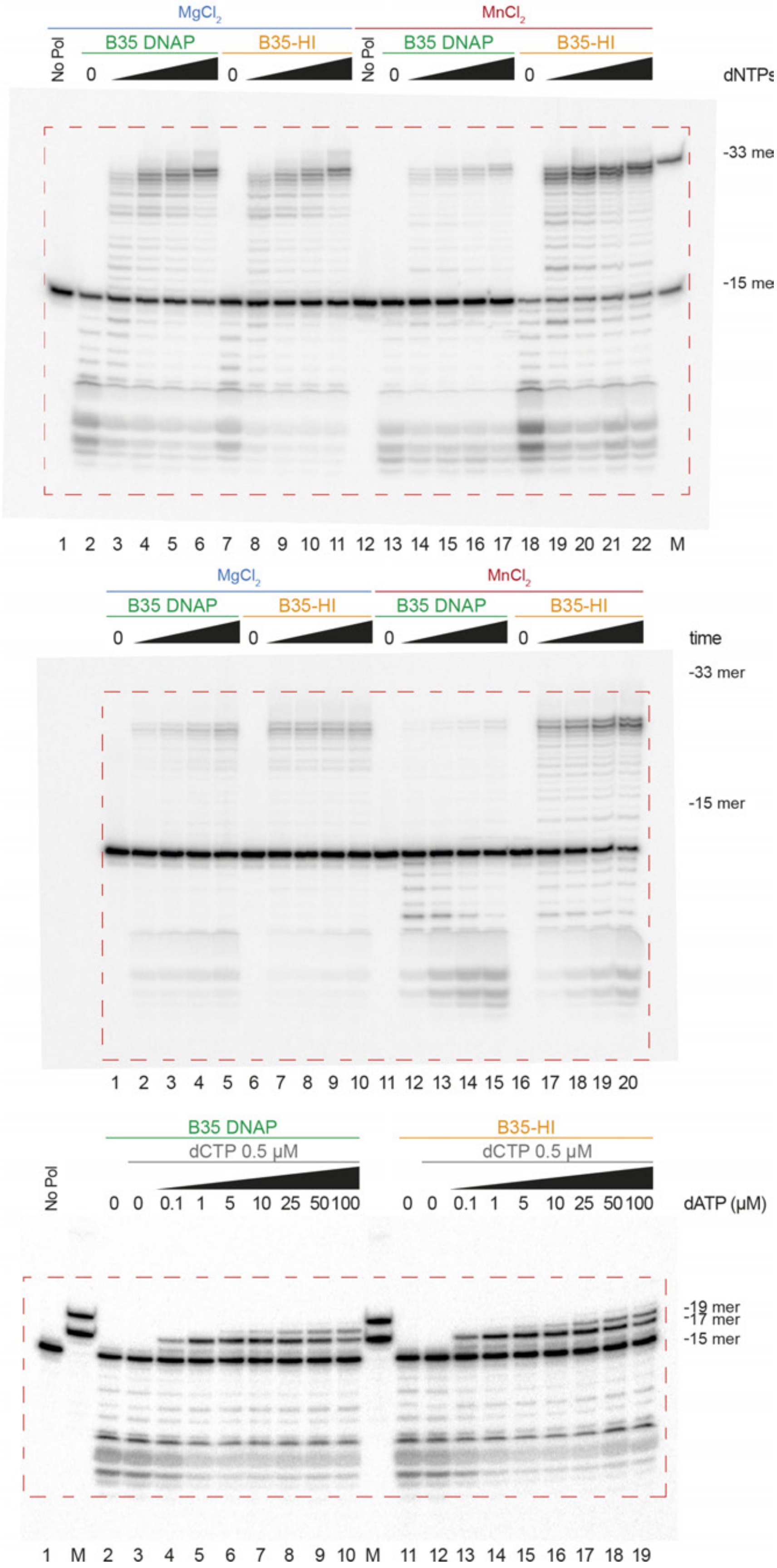

Figure S6. Uncropped version of Figure 2.

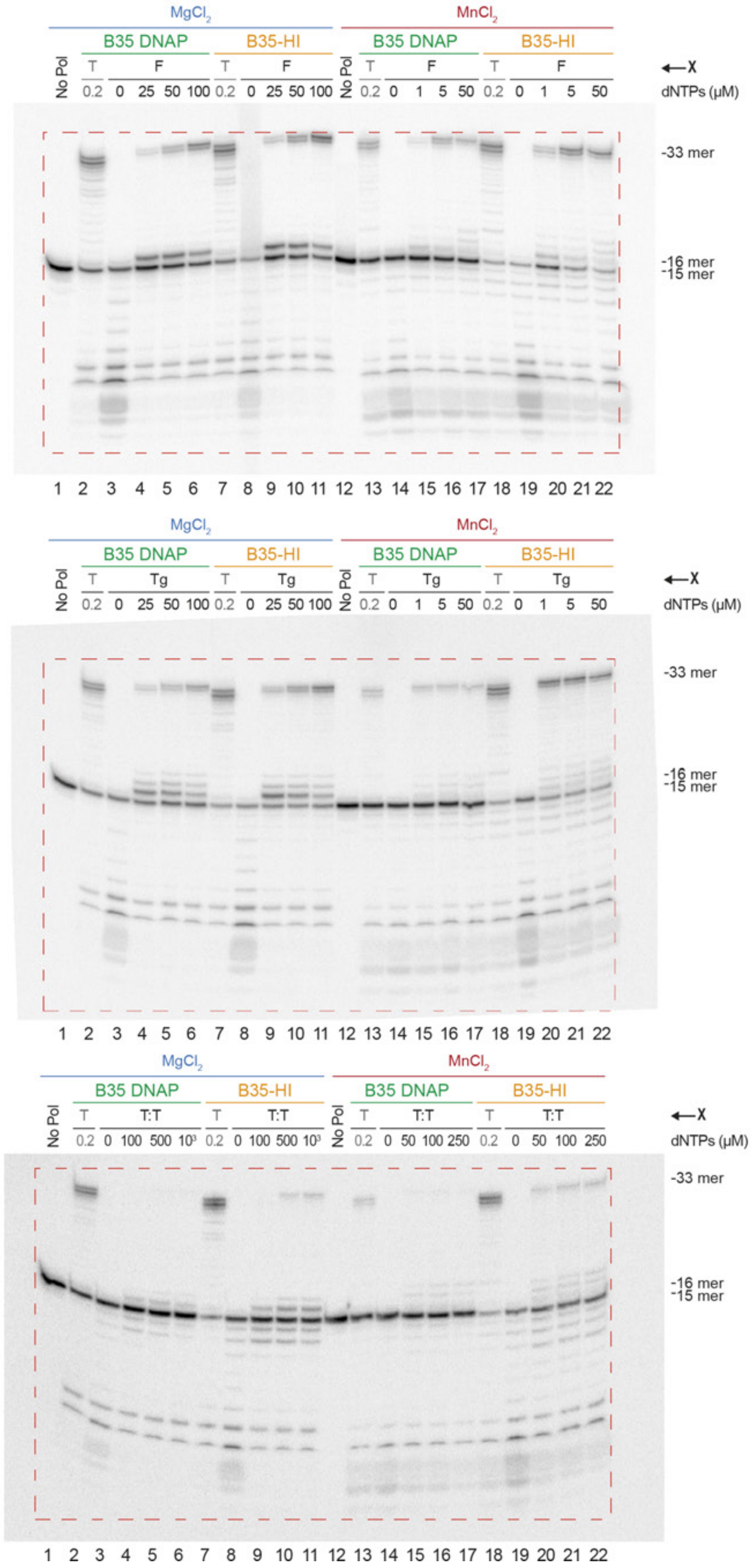

Figure S7. Uncropped version of Figure 3.

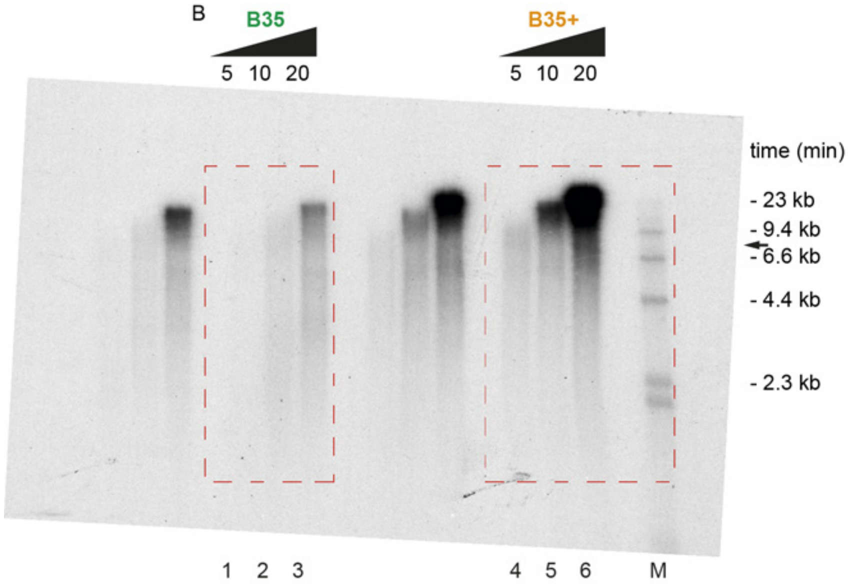

Figure S8. Uncropped version of Figure 4.

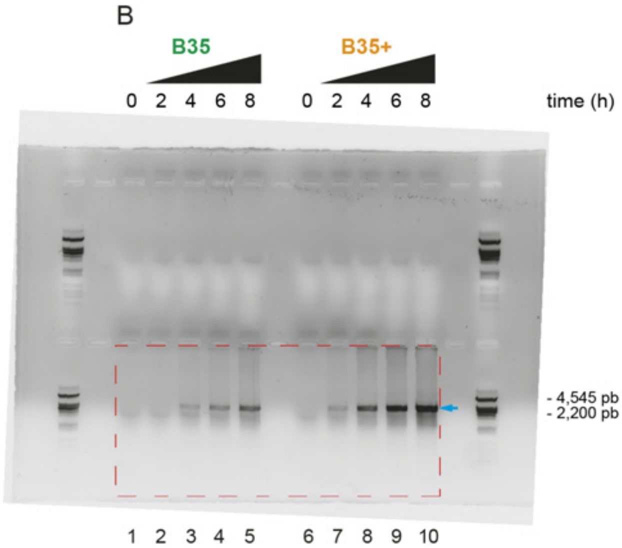

Figure S9. Uncropped version of Figure 5.

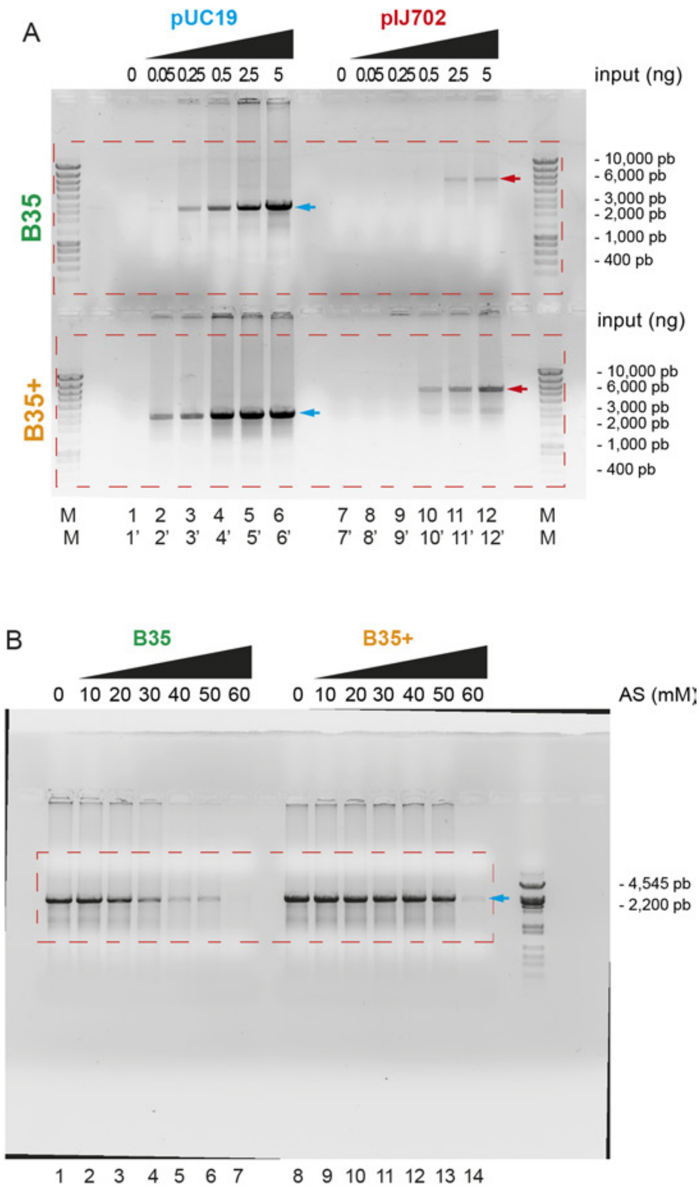

Figure S10. Uncropped version of Figure 7.

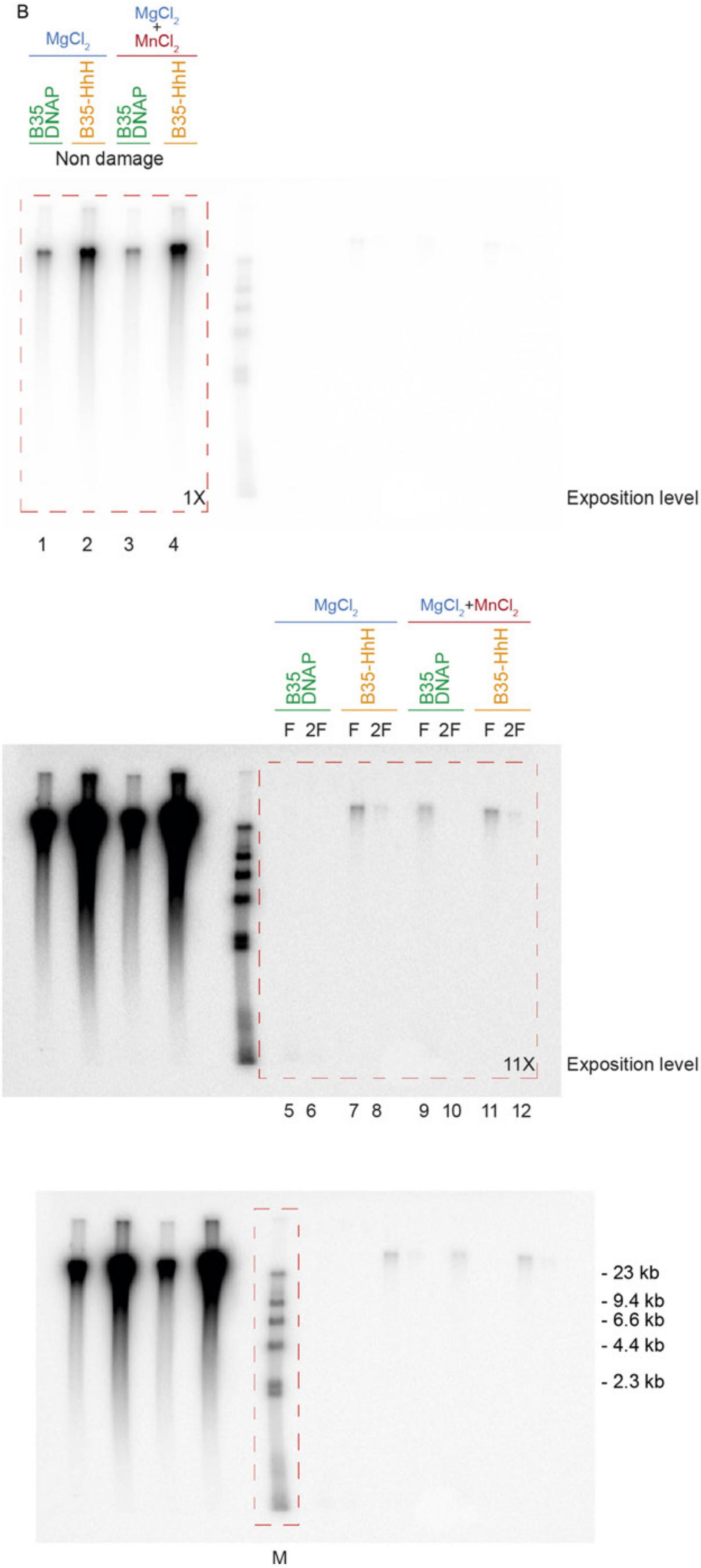

Figure S11. Uncropped version of Figure 8.

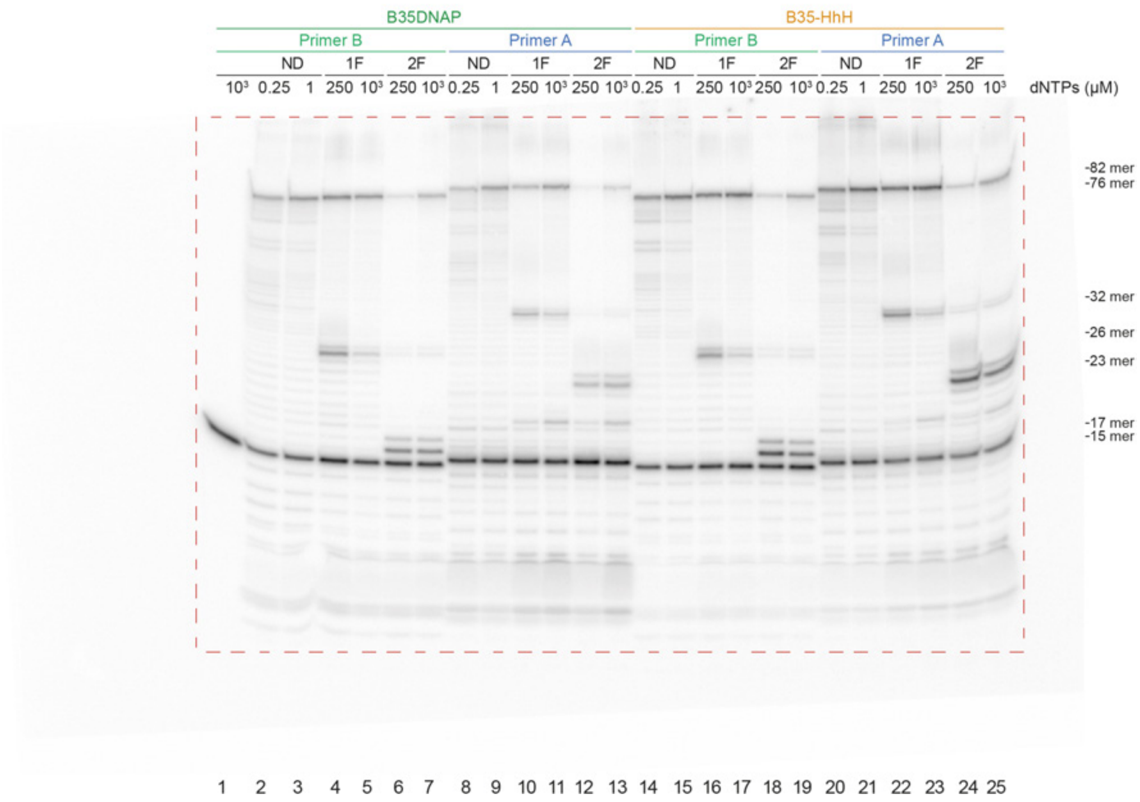

Figure S12. Uncropped version of Figure S1.

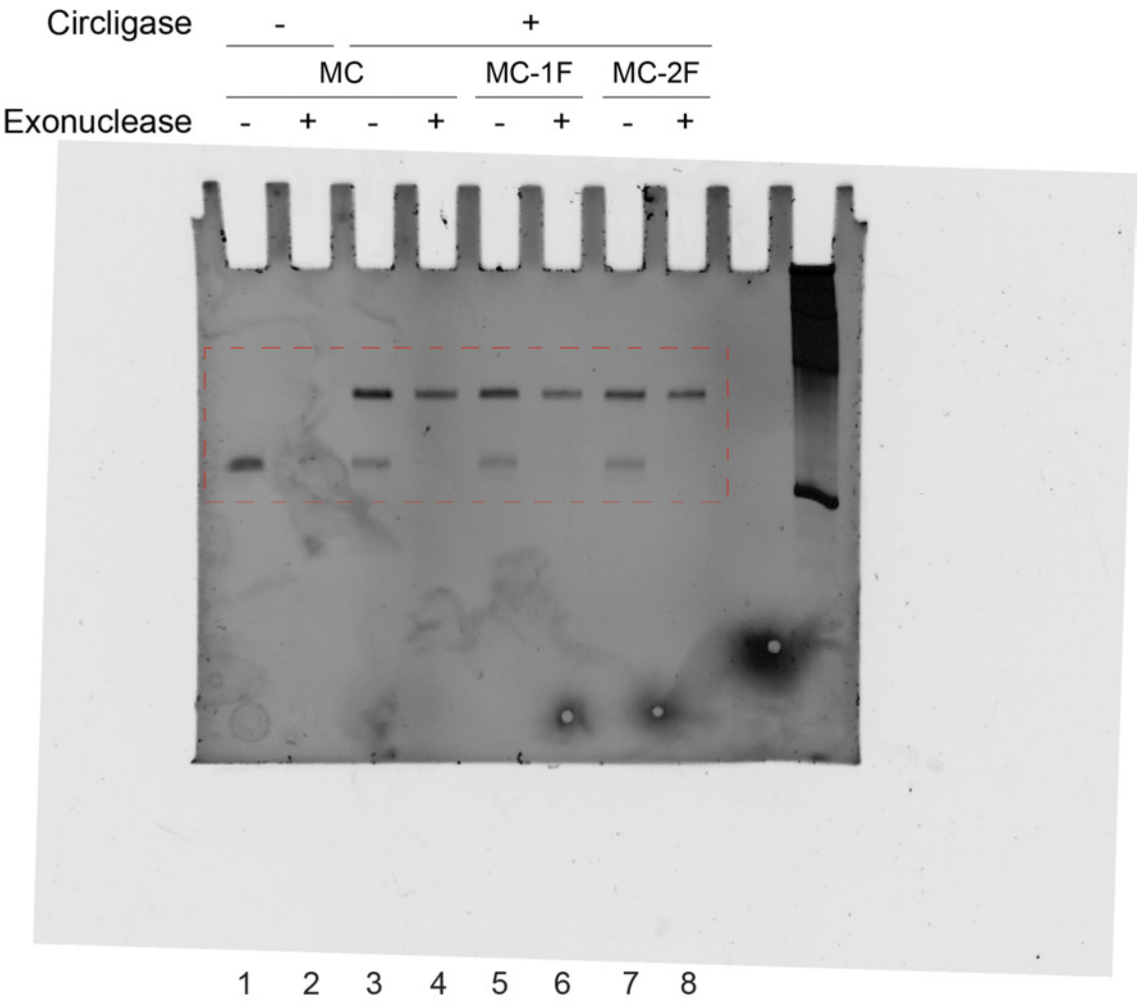

Figure S13. Uncropped version of Figure S2.

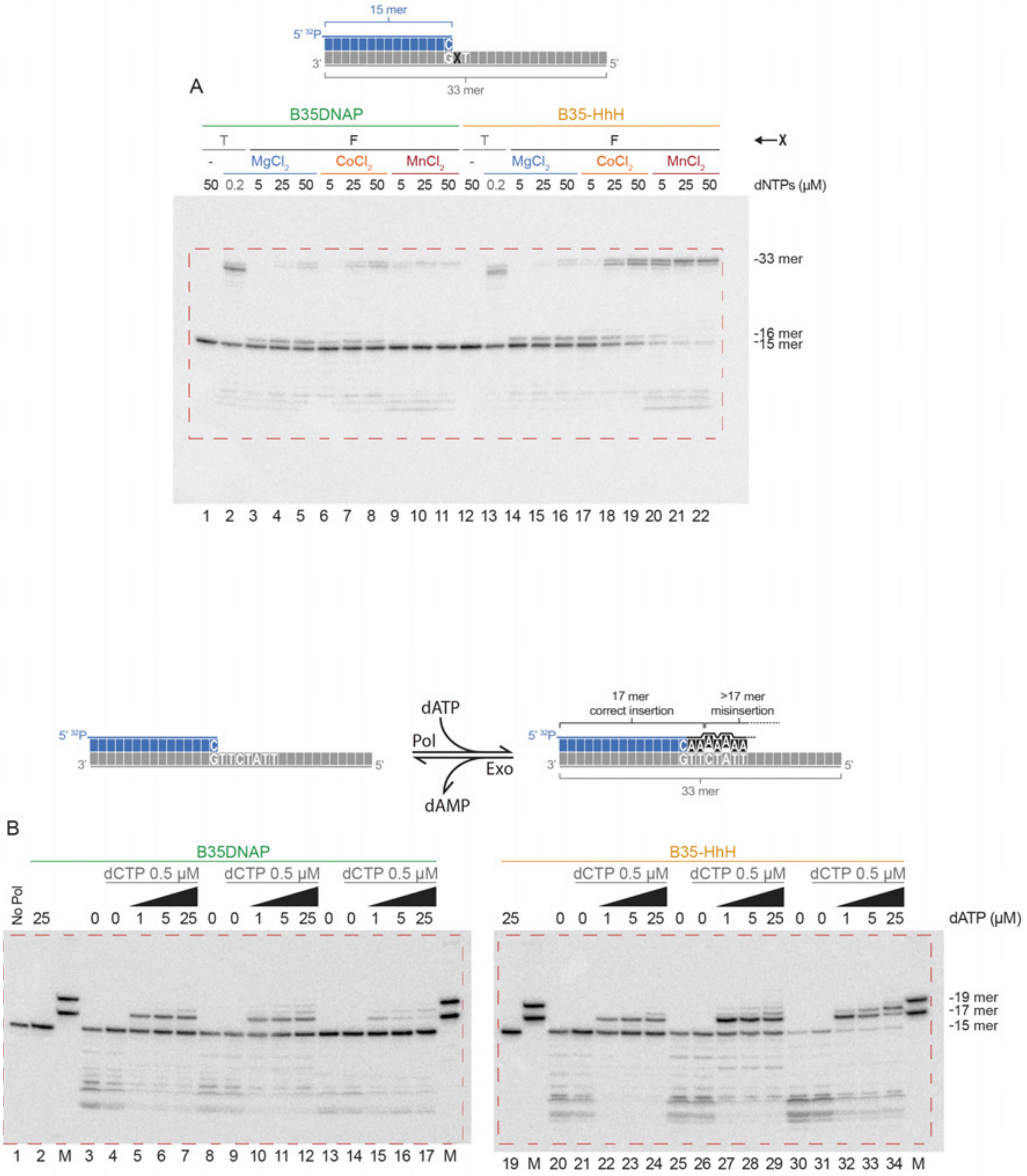

Figure S14. Uncropped version of Figure S3.

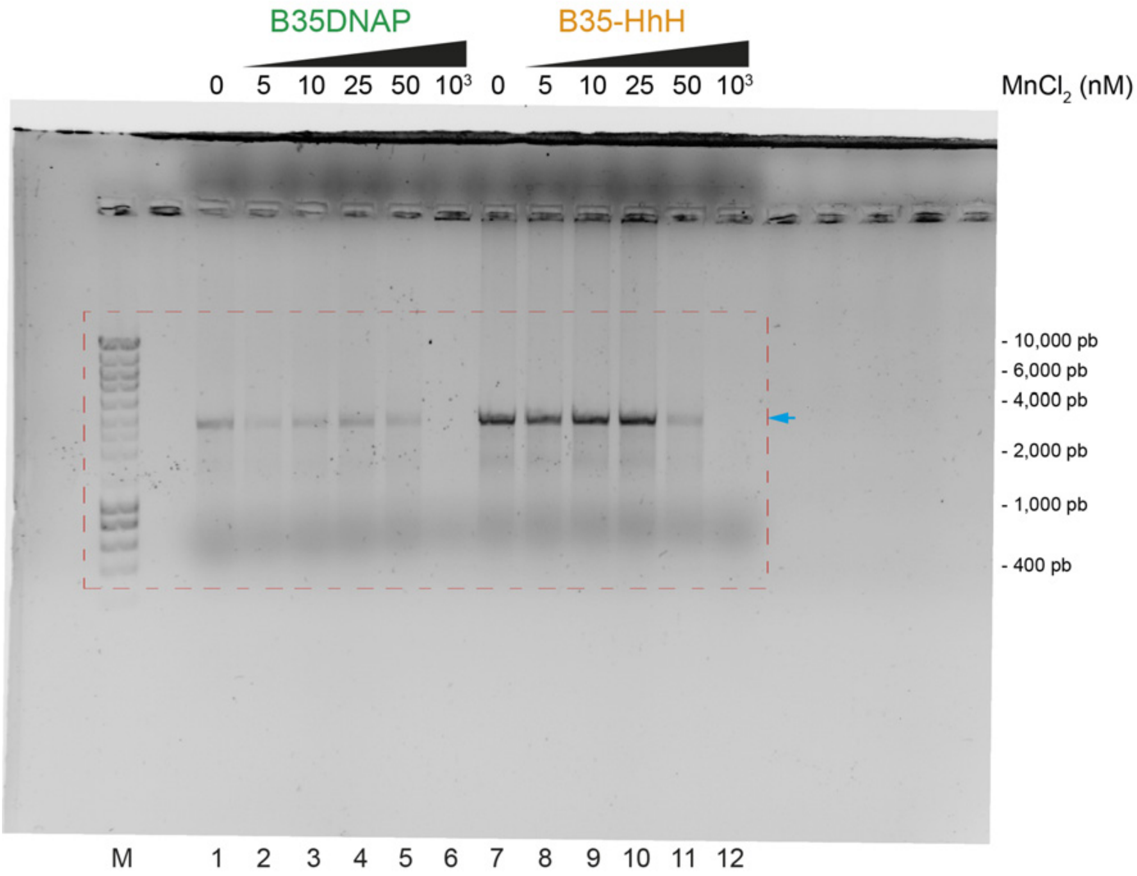

Figure S15. Uncropped version of Figure S4.

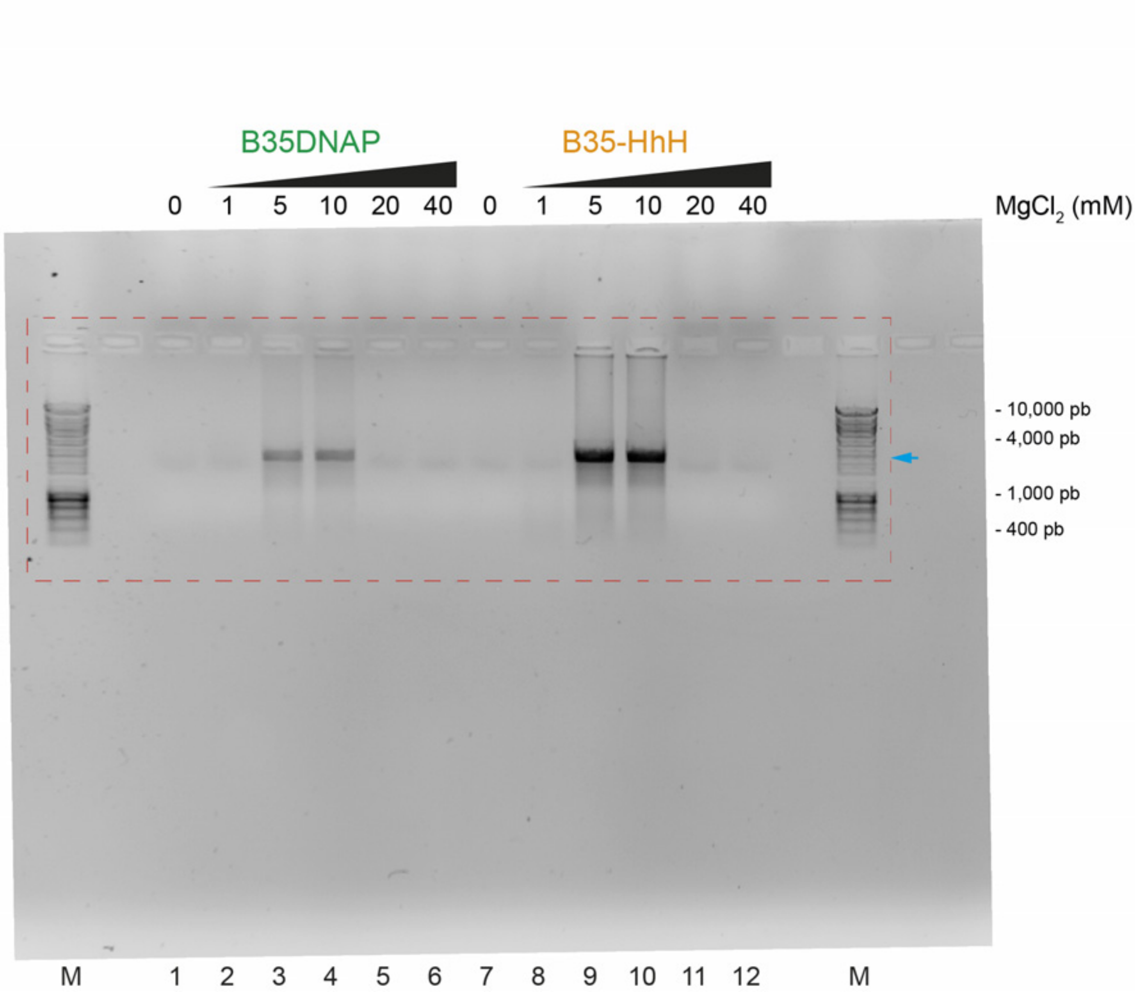

Figure S16. Uncropped version of Figure S5.

Table S1. Oligonucleotides used in this work. F stands for a tetrahydrofuran abasic site stable analog, T:T a cyclobutane thymine dimer, t a thymine glycol.

Name	Sequence (5'-3')
B35HindIII_fw	CGCGAAGCTTAAAGGAGGAAGCATATGATGACTACTACTA<br>ATAGAAAAAAGCGT
B35KasI_rev	CCGGCGCCAGAGCCGGTGCCTAAGAAACTTAATTCACCT<br>AATAGTTCTTTCAT
OL15	GATCACAGTGAGTAC
OL17	GATCACAGTGAGTACAT
OL19	GATCACAGTGAGTACATAG
OL33	GATCACAGTGAGTACAATAGAACGACGGCCAGT
OL33c	ACTGGCCGTCGTTCTATTGTACTCACTGTGATC
OL33c-THF	ACTGGCCGTCGTTCTATFGTACTCACTGTGATC
OL33c-T:T	ACTGGCCGTCGTTCTAT:TGTACTCACTGTGATC
OL33c-Tg	ACTGGCCGTCGTTCTATtGTACTCACTGTGATC
OL-M13	GTAAAACGACGGCCAGT
MC	GAATTCACTGGCCGTCGTTTTACAACGTCGTGACTGGGA<br>AAACCCTGGCGTTACCCAACTTAATCGCCTTGCAGCACAT<br>CCCCCTTTCGCCAGCTGGCGT
MC-1F	GAATTCACTGGCCGTCGTTTTACAACGTCGTGACTGGGA<br>AAACCCTGGCGFTACCCAACTTAATCGCCTTGCAGCACAT<br>CCCCCTTTCGCCAGCTGGCGT
MC-2F	GAATTCACTGGCCGTCGTTTTACAACGTCGTGACTGGGA<br>AAACCCTGGCGFTACCCAACFTAATCGCCTTGCAGCACAT<br>CCCCCTTTCGCCAGCTGGCGT
MCc-7	GGGATGTGCTGCAAG
MCc-1	TGCTGCAAGGCGATT
LacZ_sec	AGCTTGTCTGTAAGCGGATGCCG
